# Using hierarchical similarity to examine the genetics of Behçet’s disease

**DOI:** 10.1186/s13104-021-05767-6

**Published:** 2021-09-10

**Authors:** Samuel J. Shenoi, Erich J. Baker

**Affiliations:** Department of Computer Science, Baylor University, One Bear Place, Waco, TX USA

**Keywords:** Behçet syndrome, Genomics, Autoimmune disease

## Abstract

**Objective:**

Behçet’s disease (BD) is a multisystem inflammatory disease that affects patients along the historic silk road. Thus far, the pathogenesis of the disease has proved elusive due to the complex genetic interactions of the disease. In this paper, we seek to clarify the genetic factors of the disease while also uncovering other diseases of interest that present with a similar genotype as BD.

**Results:**

To do this, we employ a convergent functional genomics approach by leveraging the hierarchical similarity tool available in Geneweaver. Through our analysis, we were able to ascertain 7 BD consensus genes and 16 autoimmune diseases with genetic overlap with BD. The results of our study will inform further research into the pathogenesis of Behçet’s disease.

**Supplementary Information:**

The online version contains supplementary material available at 10.1186/s13104-021-05767-6.

## Introduction

Behçet’s disease (BD) is a multi-system inflammatory disease, demonstrating lesions of the mouth, the genitalia, and the eye [1, 2]. The disease is thought to be triggered in genetically predisposed individuals by a currently unknown combination of viral infection and environmental factors [3].

The genetics of BD are of particular interest. The HLA-B51 gene is the most closely associated risk factor for BD. However, the sizable presence of HLA-B51 negative BD patients indicates the role of at least one other genetic loci in BD pathogenesis [4, 5]. The lack of a clear mechanism of BD pathogenesis and the genetic variation present in BD patients makes it difficult to separate true genetic factors of the disease from background genetic noise.

The autoimmune nature of BD and the discovery of isolated shared genetic factors between other autoimmune diseases and BD is intriguing. For instance, ERAP1 has been shown to be a susceptibility gene in BD, psoriasis, and ankylosing spondylitis [6–8]. The molecular and genetic overlap between autoimmune diseases presents a significant area for research, as gaining a deeper understanding of the overlap can have important implications for diagnosis, treatments, and future research [9].

Addition research posits an intriguing approach towards uncovering this overlap. In addiction research, single genes rarely impact clinical phenotype, and hundreds of variants are needed to fully explain underlying genetics of the diseases. Convergent functional genomics (CFG) is leveraged to isolate important signals against this background. The premise of CFG is straight forward: the more lines of evidence for a gene, the higher it is prioritized as a gene of interest [9, 10]. This approach allows data from a variety of genomic sources to be synthesized as evidence to ascertain the impact of genes on phenotype [10].

To implement the CFG approach, we leverage the computational power of the Geneweaver Hierarchical Similarity (HiSIM) graph [11]. This tool creates a graph that is a hierarchical network of multiway geneset intersections. This enables users to find genes connected to all populated subsets of an input set of gene lists (genesets) [12]. Nodes at the top of the resulting graph contain a few strongly associated genes while nodes at the bottom contain weaker associated genes. In this work, we use the CFG approach to first find candidate genes of interest for BD and then evaluate other autoimmune diseases for genetic overlap with BD.

## Main text

### Methods

#### Data collection

Genes associated with BD were collected as genesets on Geneweaver [12]. Each of the sixteen genesets collected contained a record of BD associated genes from a single source. Eleven of the genesets originated from GWAS studies and were collected by searching the GWAS Catalog and publicly available curated genesets from Geneweaver [12, 13]. The combined GWAS data came from a global population that included samples from Iranian, Japanese, Turkish, Korean, Spanish, Western European, Middle Eastern, and Han Chinese populations. [14–17, 17–23]. One of the GWAS studies collected data regarding BD and a special type of BD that effects the GI tract, Intestinal BD (IBD) [17], and was split into two genesets for purposes of this study. The remaining BD genesets came from the NCBI gene2mesh tool [24], the Online Mendelian Inheritance in Man (OMIM) database tag “autoinflammatory, familial, Behçet’s-like” [25], Malacards [26], a BD review paper that consolidated genes of interest proposed in the literature, and a BD gene expression profile paper [27, 28]. In total, the data present in the 16 genesets, including the data consolidated by the review article geneset, came from 73 unique data sources and contained 319 genes (Additional file 1: Table S1). The union of all 16 genesets was subsequently collected using the Boolean Algebra tool on Geneweaver and stored as another geneset [12].

Publicly available Geneweaver genesets for 27 conditions were compiled in order to test BD ’s relation to other autoimmune diseases. For each of the 27 conditions, Homo Sapien genesets were found using the GeneWeaver search feature using keywords. The top 25 genesets per condition were included in the study, with priority given to manually curated genesets (Tier III) with a focused scope of study. Genesets associated with primary data (Tier I) were prioritized next, followed by derivative (Tier II) data sets. For example, a tier 1 geneset documenting a psoriasis GWAS study was given preference over a tier 2 study documenting a psoriasis and Crohn’s disease GWAS study. Disease genesets were created by using the Geneweaver Boolean Algebra tool on publicly available curated genesets (Additional file 1: Table S2) [12]. These 27 genesets were the product of 309 unique and individual genesets spanning over 4000 genes created using the Boolean Algebra tool on Geneweaver [11]

#### BD HiSIM run

To find BD consensus genes, the Geneweaver HiSIM graph was run with the parameters reported in Additional file 1: Table S3 and with the 16 collected BD genesets as input.

#### Autoimmune HiSIM run

To determine the genetic overlap between BD and other autoimmune diseases, the HiSIM graph tool was run with the parameters documented in Additional file 1: Table S3 on 27 autoimmune disease genesets constructed using the Boolean Algebra tool and the BD Union geneset. The data from the Autoimmune HiSIM run was then filtered to find relevant connections between BD and other autoimmune diseases.

#### Jaccard geneset analysis

A Jaccard geneset analysis was conducted on autoimmune diseases identified with a high BD genetic similarity to determine the statistical significance of the overlap. The Jaccard geneset analysis was run using the Geneweaver Jaccard Similarity tool [12].

#### Neighbor joining analysis

In order to find the top five most genetically similar diseases to BD, a neighbor joining tree was created using the ape R package [29]. The distance between any two diseases was defined using the normalized Jaccard formula seen in Formula 1.

1 $$\begin{aligned} 1 - \frac{A \cap B}{min(size(A), size(B))}. \end{aligned}$$

Formula 1 took into account the differences in geneset size between two genesets when determining their overlap. By taking the size of the intersection between the two sets and dividing it by the largest possible overlap that could occur between the two sets, the formula allowed sets of different sizes to be compared. The distance between all possible pair combinations of the identified diseases was calculated and stored in a distance matrix. The distance matrix was used as input to the ape neighbor joining program [29, 30].

## Results

### BD HiSIM run

**Fig. 1:**
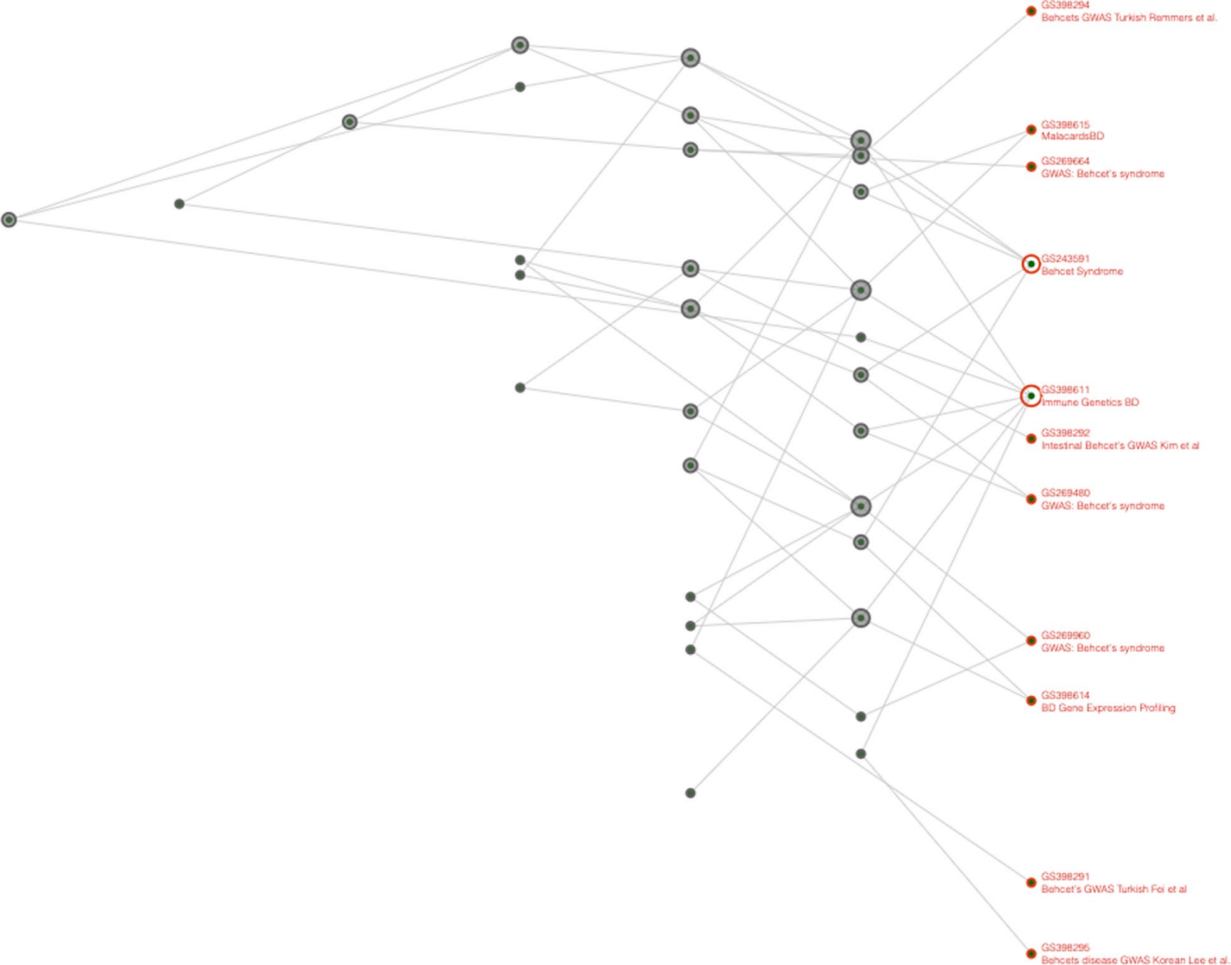
HiSIM graph between 17 BD gensets

Running the HiSIM graph on the BD collected genesets resulted in the graph shown in Fig. 1. HLA-B was identified in 7 genesets making it the most common gene among all tested genes. It was followed by IL-10 in 6 genesets, IL23R in 5 genesets, and HLA-A, STAT4, MICA, and ERAP1 in 4 genesets (Additional file 1: Table S4).

### Autoimmune disease run

**Fig. 2:**
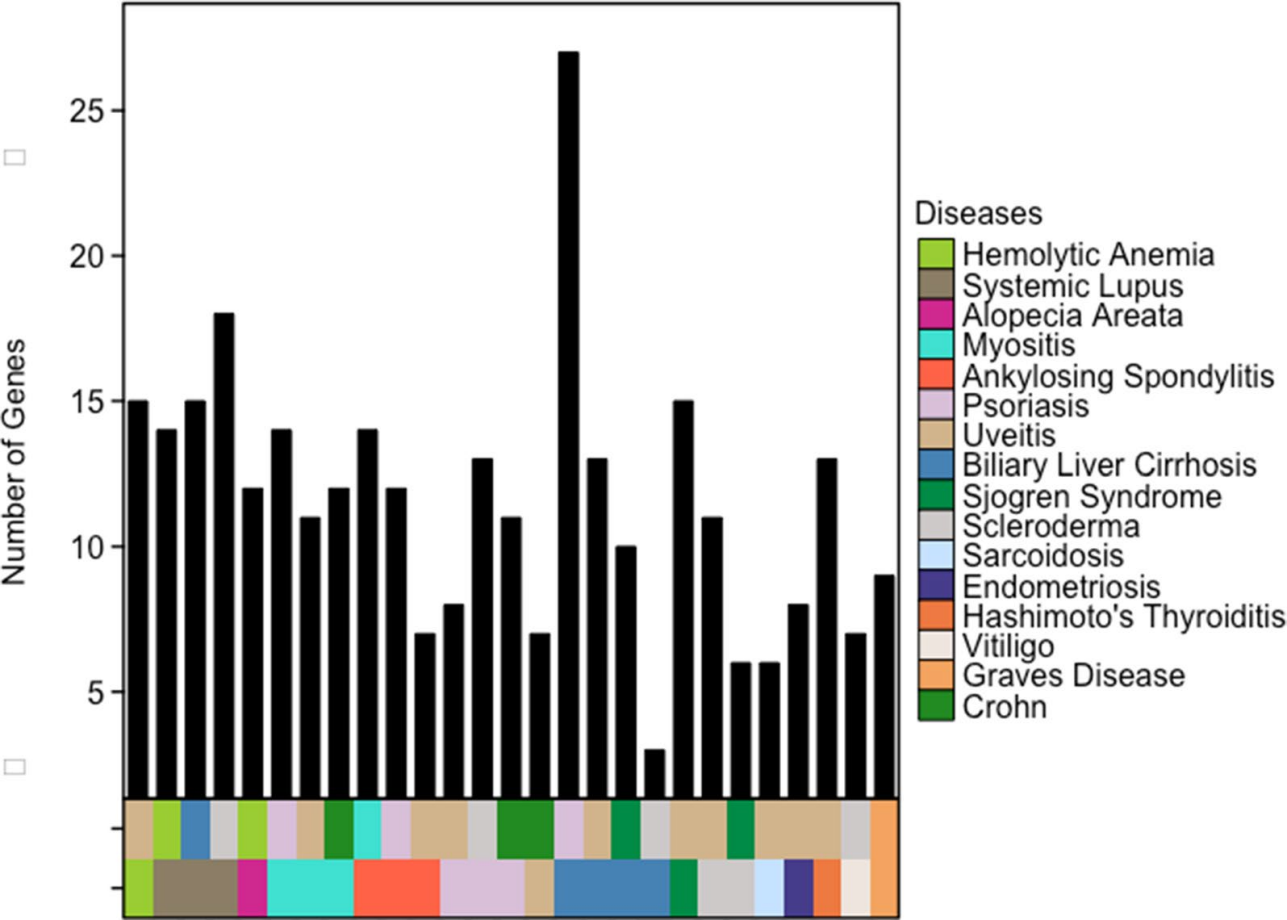
Graph displaying identified HiSIM nodes with the largest gene overlap with BD. 16 conditions were found to have some overlap with BD out of the 27 tested

After collecting autoimmune genesets on Geneweaver, the HiSIM graph was run on 27 unique autoimmune diseases and BD. Figure 2 displays the genetic overlap between the autoimmune diseases and BD as determined by the HiSIM graph. Out of the 27 conditions tested, 16 were found to have some genetic overlap with BD and were subsequently used to preform the Jaccard Analysis.

### Jaccard geneset analysis

Using the 16 identified conditions from the Autoimmune HiSIM run, a Jaccard Geneset Analysis was run. The results from the Jaccard geneset analysis found no statistically significant overlap between any of the tested pairs of conditions (Additional file 1: Figure S1).

### Neighbor joining analysis

**Fig. 3:**
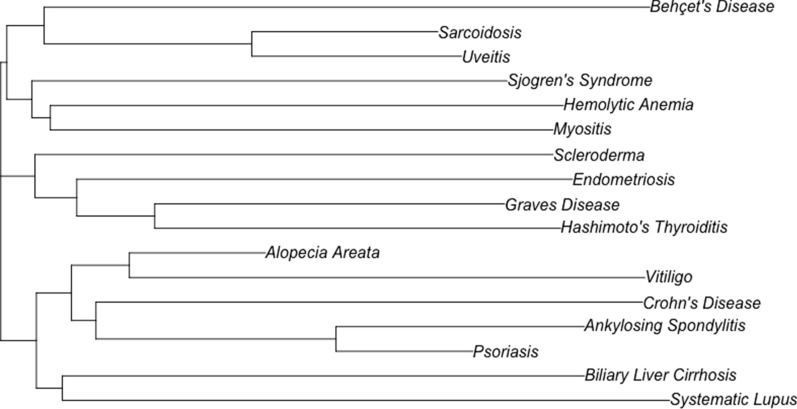
Results of the neighbor joining tree

The genetic overlap between BD and the 16 identified autoimmune diseases was then normalized using Formula 1 and used as input for the Neighbor Joining Tree (Fig. 3). The tree indicates that BD is closest to Sarcoidosis, Uveitis, Sjögren’s Syndrome, Hemolytic Anemia, and Myositis.

## Discussion

The results of the BD HiSIM graph run are consistent with previous BD research. All top scoring genes have previously been identified as associated with BD [6, 27, 31]. The emergence of HLA-B as the top scoring gene is unsurprising given that HLA-B51 is the most closely associated risk factor of BD [4, 31, 32].

The results of the BD autoimmune disease run identified 16 diseases with a BD genetic overlap. However, these associations were unable to achieve statistical significance based on the results of our Jaccard Similarity run (Additional file 2: Figure S1). To account for this, we proposed a modified Jaccard similarity formula (Formula 1) and constructed a neighbor joining tree that allowed us to quantify the genetic similarity of each of the 16 identified diseases to BD.

The results of our CFG approach have the benefit of identifying autoimmune diseases with a shared genetic architecture to BD. The inclusion of certain diseases in the 16 identified diseases was expected: Uveitis is symptom of BD [32], Sjögren’s Syndrome often presents with but is not associated with BD [33–35], and Crohn’s disease presents with similar symptoms to IBD [36].

The inclusion of psoriasis in our list was noteworthy. Psoriatic arthritis has been documented to sometimes be confused with BD articular involvement [37]. Recently, Hahn *et al.* found that psoriasis patients were twice as likely to be diagnosed with BD compared to controls [38].

Encouragingly, there have been documented case reports of patients presenting simultaneously with both BD and one of conditions identified in this study [39–53]. The only exception to this was Alopecia Areata which to our knowledge has not been reported as presenting simultaneously with BD. These findings, coupled with our genetic analysis, might provide evidence for an underlying genetic mechanism related to the pathogenesis of these diseases. It will be left up to future work to fully clarify these relationships.

## Conclusion

Behçet’s disease is a complex multi-system inflammatory disease whose exact pathogenesis continues to elude researchers [2, 3]. Genetically, the identification of HLA-B51 as a major, but not sole, susceptibility gene has led to the hunt for other genetic factors of the disease [4]. The resulting identification of multiple genes of interest, however, does not explicitly establish the contributions of each of these genes towards the overall presentation of the disease [6, 7, 27, 32, 54–57]. Interestingly, research into some of these genes has uncovered roles of these genes in the progression of other autoimmune diseases [6–8, 58]. Therefore, researching autoimmune diseases in relation to other autoimmune diseases might be needed to fully understand human disease.

In this study, we employed a functional convergent genomics approach to discover (1) the genetic factors and (2) related autoimmune conditions of Behçet’s disease. The power of this approach lies in its ability to synthesize information from multiple genomics data sources [9] and in its recognition of shared genetic factors of autoimmune diseases [6, 7, 56]. The Geneweaver HiSIM graph’s ability to synthesize this information presents a valuable opportunity for further discovery in this line of research [11]. Furthermore, our results using this approach confirmed existing BD genetics research and identified 16 autoimmune diseases that share an underlying genetic relationship to BD. Almost all of these associations have documented clinical findings linking them with BD, further providing evidence towards our results. It will be left up to further research to fully uncover the complex genetic interactions underlying these diseases and the shared genetic mechanisms between them.

## Limitations

A limitation to our study was that our selection criteria excluded non-autoimmune conditions, such as oral cavity cancer, that might mimic BD phenotype presentations. Another limitation to this study was that the Jaccard Similarity test used suffers from dataset size limitations [59].

## Supplementary Information

**Additional file 1**:** Table S1** contains a list of the 27 autoimmune diseases tested,the number of genesets collected for each condition, the total number of genes collected for condition, and theassociated publications for the genesets.** Table S2** contains the parameters used to run the HiSIM graph.** Table S3**contains the top results from the BD HiSIM run.** Table S4** contains the sources used to create the review articlegeneset used for the BD HiSIM run.

**Additional file 2: Figure S1** shows the results from the Jaccard Similarity run.

**Additional file 3:** The genesets included in this text (.txt) file reference studies looking into the genetics of BD. These genesets wereused to conduct the BD HiSIM Run. This text file was created using the export genesets functionality inGeneweaver. All genesets included are publicly available and searchable on Geneweaver.

**Additional file 4:** The genesets included in this text (.txt) file were created using the Boolean Algebra tool on publicly availablegenesets on Geneweaver. These genesets were used to conduct the BD Auotimmune Disease Run. This text file wascreated using the export genesets functionality in Geneweaver. All genesets included are publicly available andsearchable on Geneweaver.

## Data Availability

The genesets used for this study were exported from Geneweaver and included as Additional files 3 and 4.

## Abbreviations

BD: Behçet’s disease
CFG: Convergent functional genomics
