# Supplementary material for: Using hierarchical similarity to examine the genetics of Behçet’s disease
Source: BMC Res Notes. 2021 Sep 10;14:353. doi: 10.1186/s13104-021-05767-6 (PMC8434716; doi:10.1186/s13104-021-05767-6)
Supplement: Supplementary file 1 — Additional file 1: Table S1 contains a list of the 27 autoimmune diseases tested,the number of genesets collected for each condition, the total number of genes collected for condition, and theassociated publications for the genesets. Table S2 contains the parameters used to run the HiSIM graph. Table S3contains the top results from the BD HiSIM run. Table S4 contains the sources used to create the review articlegeneset used for the BD HiSIM run. [file 13104_2021_5767_MOESM1_ESM.pdf]

Genset	Num Genes	Sources
OMIM Geneset	1	[1]
NCBI gene2mesh Geneset	67	[2]
Malacards Geneset	10	[3]
Gene Expression Profile Geneset	145	[4]
GWAS in Korean population	3	[5]
GWAS in Turkish population	5	[6]
GWAS in Spanish European population	2	[7]
GWAS in Korean population	1	[8]
IBD GWAS in Korean population	4	[8]
GWAS in Japanese population	4	[9]
GWAS in Turkish population	8	[10]
GWAS in Iranian population	2	[11]
GWAS in Chinese population	9	[12]
GWAS in Western Europeans, Middle Eastern and Turkish individuals	2	[13]
GWAS in Turkish population	3	[14]
Deng Review Article Geneset [15]	31	[16; 17; 18; 19; 20; 21; 22; 23; 24; 25; 26; 27; 28; 29; 30; 31; 12; 32; 33; 34; 35; 36; 13; 10; 37; 38; 39; 5; 40; 41; 42; 43; 44; 45; 46; 47; 9; 48; 49; 50; 51; 7; 52; 53; 54; 55; 56; 57; 58; 59; 14; 60; 61; 62; 63; 64; 65; 66; 67; 68; 11; 69; 70; 71; 72; 73]

Table S1: Sources used to create BD genesets

Condition	Number of Genes	Number of Genesets	Publications
Achalasia	12	3	[74]
Vitiligo	107	10	[75; 76; 77; 78; 79; 80; 81]
Celiac Disease	428	22	[82; 83; 84; 85; 86; 87; 88; 89; 74; 90; 91; 92; 93; 94; 95]
Crohn's Disease	273	25	[96; 97; 98; 99; 87; 100; 101; 102; 103; 104; 105; 106; 107; 108; 109; 110; 111; 112; 113]
Psoriasis	382	23	[114; 115; 116; 116; 117; 118; 119; 120; 121; 122; 123; 124; 125; 126; 127; 128]
Diabetes	130	20	[129; 130; 131; 132; 133; 134; 135; 136; 137; 138; 139; 140; 141; 142; 143; 144; 145; 146; 147; 148]
Uveitis	130	7	[74]
Sarcoidosis	107	7	[149; 100; 150]
Myositis	82	5	[74; 151]
Scleroderma	148	9	[152; 153; 74; 154; 155]
Agammaglobulinemia	39	3	[74]
Alopecia Areata	26	3	[156]
Restless Leg Syndrome	27	6	[74; 157; 158; 159; 160]
Sjögren's Syndrome	145	7	[161; 139; 162; 163]
Hashimoto's Thyroiditis	99	6	[74; 164; 165]
Addison Disease	40	4	[166]
Amyloidosis	58	12	[74]
Ankylosing Spondylitis	132	6	[167; 168; 90; 169]
Chagas Syndrome	159	7	[170]
MS	201	25	[171; 172; 173; 174; 175; 176; 177; 178; 179; 180; 181; 182; 183; 184; 185; 186; 187; 188; 189; 190; 191; 192; 193; 194]
Endometriosis	226	9	[195; 196; 74; 197; 198; 199; 200]
Ulcerative Colitis	386	23	[201; 202; 203; 204; 205; 206; 100; 207; 103; 208; 209; 210; 211; 108; 212; 213; 214; 215; 216; 217; 218]
Hemolytic Anemia	261	20	[74; 219]
Systematic Lupus	269	25	[220; 221; 222; 223; 161; 224; 225; 226; 227; 228; 229; 230; 231; 232; 233; 234; 154; 235; 236; 237; 238; 239]
Liver Cirrhosis	130	8	[240; 241; 242; 74; 243; 244; 245]
Graves' Disease	121	8	[246; 247; 248; 74; 249; 165; 250]
IgA nephropathy	39	6	[251; 252; 253; 254; 255; 256]

Table S2: Genetic information from 27 autoimmune diseases were collected from multiple publicly available genesets on Geneweaver. Genesets from each condition were then consolidated into one geneset using the Boolean Algebra tool.

**Parameter**	**Value**
Disable Bootstrap	False
Node Cutoff	Auto
Homology	Included
Genes In Node	All
Use FDR	False
Hide Unemphasized	False
p-value	1
Minimum Overlap	1%
Minimum Genes	1
Permutation Time Limit	5
Max In Node	4
Permutations	0
Max Level	40

Table S3: Parameters used for both runs of the Geneweaver HiSIM graph

Num Genesets	Gene	Genesets
7	HLA-B	Behçet's GWAS [8], BD Gene2Mesh, Behçet's GWAS [7], Behçet's GWAS [11], Malacards BD [3], Immune Genetics of BD [15], Behçet's GWAS [14]
6	IL-10	Intestinal Behçet's GWAS[8], Behçet's GWAS [14], BD Gene2Mesh, Behçet's GWAS [9], Malacards BD [3], Immune Genetics of BD [15]
5	IL23R	BD Gene2Mesh, Behçet's GWAS [14], Malacards BD [3], Immune Genetics of BD [15], Behçet's GWAS [9]
4	HLA-A	BD Gene2Mesh, Behçet's GWAS [7], Behçet's GWAS [14], Immune Genetics of BD [15]
4	STAT4	Immune Genetics of BD [15], BD Gene2Mesh, Behçet's GWAS [12], Behçet's GWAS [257]
4	ERAP1	Intestinal Behçet's GWAS[8], Malacards BD [3], Behçet's GWAS [257], Immune Genetics of BD [15]
4	MICA	BD Gene2Mesh, Behçet's GWAS [12], Immune Genetics of BD [15], Behçet's GWAS [14]

Table S4: Abbreviated results from the BD HiSIM run. IL10 was identified in 7 genesets making it the most common gene amongst all tested genes. HLA-B was the next most common gene and was found in 6 genesets. IL23R was the third most common gene; it was found in 5 genesets. Finally, HLA-A, STAT4, MICA, and ERAP1 were all found in 4 genesets.

References

[1] Amberger JS, Bocchini CA, Scott AF, Hamosh A. OMIM.org: leveraging knowledge across phenotype–gene relationships. Nucleic Acids Research. 2019 Jan;47(D1):D1038–D1043. Available from: https://academic.oup.com/nar/article/47/D1/D1038/5184722.

[2] Athey BD, Cavalcoli JD, Jagadish HV, Omenn GS, Mirel B, Kretzler M, et al. The NIH National Center for Integrative Biomedical Informatics (NCIBI). Journal of the American Medical Informatics Association. 2012 Mar;19(2):166–170. Available from: https://academic.oup.com/jamia/article-lookup/doi/10.1136/amiajnl-2011-000552.

[3] Rappaport N, Nativ N, Stelzer G, Twik M, Guan-Golan Y, Iny Stein T, et al. MalaCards: an integrated compendium for diseases and their annotation. Database. 2013 Jan;2013. Available from: https://academic.oup.com/database/article/doi/10.1093/database/bat018/331798.

[4] Puccetti A, Fiore PF, Pelosi A, Tinazzi E, Patuzzo G, Argentino G, et al. Gene Expression Profiling in Behcet's Disease Indicates an Autoimmune Component in the Pathogenesis of the Disease and Opens New Avenues for Targeted Therapy. Journal of Immunology Research. 2018;2018:1–18. Available from: https://www.hindawi.com/journals/jir/2018/4246965/.

[5] Lee YJ, Horie Y, Wallace GR, Choi YS, Park JA, Choi JY, et al. Genome-wide association study identifies GIMAP as a novel susceptibility locus for Behçet's disease. Annals of the Rheumatic Diseases. 2013 Sep;72(9):1510–1516. Available from: http://ard.bmj.com/lookup/doi/10.1136/annrheumdis-2011-200288.

[6] Fei Y, Webb R, Cobb BL, Direskeneli H, Saruhan-Direskeneli G, Sawalha AH. Identification of novel genetic susceptibility loci for Behçet's disease using a genome-wide association study. Arthritis Research & Therapy. 2009;11(3):R66.

[7] Ortiz-Fernández L, Carmona FD, Montes-Cano MA, García-Lozano JR, Conde-Jaldón M, Ortego-Centeno N, et al. Genetic Analysis with the Immunochip Platform in Behçet Disease. Identification of Residues Associated in the HLA Class I Region and New Susceptibility Loci. PloS One. 2016;11(8):e0161305.

[8] Kim SW, Jung YS, Ahn JB, Shin ES, Jang HW, Lee HJ, et al. Identification of genetic susceptibility loci for intestinal Behçet's disease. Scientific Reports. 2017;7:39850.

[9] Mizuki N, Meguro A, Ota M, Ohno S, Shiota T, Kawagoe T, et al. Genome-wide association studies identify IL23R-IL12RB2 and IL10 as Behçet's disease susceptibility loci. Nature Genetics. 2010 Aug;42(8):703–706.

[10] Kirino Y, Nakajima H. Clinical and Genetic Aspects of Behçet's Disease in Japan. Internal Medicine. 2019 May;58(9):1199–1207. Available from: https://www.jstage.jst.go.jp/article/internalmedicine/58/9/58_2035-18/_article.

[11] Xavier JM, Shahram F, Sousa I, Davatchi F, Matos M, Abdollahi BS, et al. *FUT2* : filling the gap between genes and environment in Behçet's disease? Annals of the Rheumatic Diseases. 2015 Mar;74(3):618–624. Available from: http://ard.bmj.com/lookup/doi/10.1136/annrheumdis-2013-204475.

[12] Hou S, Yang Z, Du L, Jiang Z, Shu Q, Chen Y, et al. Identification of a susceptibility locus in STAT4 for Behçet's disease in Han Chinese in a genome-wide association study. Arthritis & Rheumatism. 2012;64(12):4104–4113. Available from: https://onlinelibrary.wiley.com/doi/abs/10.1002/art.37708.

[13] Kappen JH, Medina-Gomez C, van Hagen PM, Stolk L, Estrada K, Rivadeneira F, et al. Genome-wide association study in an admixed case series reveals IL12A as a new candidate in Behçet disease. PloS One. 2015;10(3):e0119085.

[14] Remmers EF, Cosan F, Kirino Y, Ombrello MJ, Abaci N, Satorius C, et al. Genome-wide association study identifies variants in the MHC class I, IL10, and IL23R-IL12RB2 regions associated with Behçet's disease. Nature Genetics. 2010 Aug;42(8):698–702.

[15] Deng Y, Zhu W, Zhou X. Immune Regulatory Genes Are Major Genetic Factors to Behcet Disease: Systematic Review. The Open Rheumatology Journal. 2018 Jun;12(1):70–85. Available from: https://openrheumatologyjournal.com/VOLUME/12/PAGE/70/.

[16] Arbour NC, Lorenz E, Schutte BC, Zabner J, Kline JN, Jones M, et al. TLR4 mutations are associated with endotoxin hyporesponsiveness in humans. Nature Genetics. 2000 Jun;25(2):187–191.

[17] Ben Ahmed M, Houman H, Abdelhak S, Ben Ghorbel I, Miled M, Dellagi K, et al. MICA transmembrane region polymorphism and HLA B51 in Tunisian Behçet's disease patients. Advances in Experimental Medicine and Biology. 2003;528:225–228.

[18] Ben Dhifallah I, Lachheb J, Houman H, Hamzaoui K. Toll-like-receptor gene polymorphisms in a Tunisian population with Behçet's disease. Clinical and Experimental Rheumatology. 2009 Apr;27(2 Suppl 53):S58–62.

[19] Carapito R, Shahram F, Michel S, Le Gentil M, Radosavljevic M, Meguro A, et al. On the genetics of the Silk Route: association analysis of HLA, IL10, and IL23R-IL12RB2 regions with Behçet's disease in an Iranian population. Immunogenetics. 2015 Jun;67(5-6):289–293. Available from: http://link.springer.com/10.1007/s00251-015-0841-6.

[20] Chae JJ, Wood G, Masters SL, Richard K, Park G, Smith BJ, et al. The B30.2 domain of pyrin, the familial Mediterranean fever protein, interacts directly with caspase-1 to modulate IL-1beta production. Proceedings of the National Academy of Sciences of the United States of America. 2006 Jun;103(26):9982–9987.

[21] Chang JT, Shevach EM, Segal BM. Regulation of interleukin (IL)-12 receptor beta2 subunit expression by endogenous IL-12: a critical step in the differentiation of pathogenic autoreactive T cells. The Journal of Experimental Medicine. 1999 Mar;189(6):969–978.

[22] Choy MK, Phipps ME. MICA polymorphism: biology and importance in immunity and disease. Trends in Molecular Medicine. 2010 Mar;16(3):97–106.

[23] Cohen R. Association of the MIC-A gene and HLA-B51 with Behcet's disease in Arabs and non-Ashkenazi Jews in Israel. Annals of the Rheumatic Diseases. 2002 Feb;61(2):157–160. Available from: https://ard.bmj.com/lookup/doi/10.1136/ard.61.2.157.

[24] Esmaeili M, Bonyadi M, Khabbazi A, Ebrahimi AA, Sharif SK, Hajialilo M, et al. Common MEFV mutations in Iranian Azeri Turkish patients with Behçet's disease. Scandinavian Journal of Rheumatology. 2011;40(5):383–386.

[25] Fang J, Hu R, Hou S, Ye Z, Xiang Q, Qi J, et al. Association of TLR2 gene polymorphisms with ocular Behcet's disease in a Chinese Han population. Investigative Ophthalmology & Visual Science. 2013 Dec;54(13):8384–8392.

[26] Filén JJ, Filén S, Moulder R, Tuomela S, Ahlfors H, West A, et al. Quantitative proteomics reveals GIMAP family proteins 1 and 4 to be differentially regulated during human T helper cell differentiation. Molecular & cellular proteomics: MCP. 2009 Jan;8(1):32–44.

[27] Giza M, Koftori D, Chen L, Bowness P. Is Behçet's disease a 'class 1-opathy'? The role of HLA-B*51 in the pathogenesis of Behçet's disease. Clinical and Experimental Immunology. 2018;191(1):11–18.

[28] González-Escribano MF, Rodríguez MR, Aguilar F, Alvarez A, Sanchez-Roman J, Núñez-Roldán A. Lack of association of MICA transmembrane region polymorphism and Behçet's disease in Spain: MICA-TM alleles in Behçet's disease. Tissue Antigens. 1999 Sep;54(3):278–281. Available from: `http://doi.wiley.com/10.1034/j.1399-0039.1999.540309.x`.

[29] Holm SJ, Carlén LM, Mallbris L, Ståhle-Bäckdahl M, O'Brien KP. Polymorphisms in the SEEK1 and SPR1 genes on 6p21.3 associate with psoriasis in the Swedish population. Experimental Dermatology. 2003 Aug;12(4):435–444.

[30] Holtschke T, Löhler J, Kanno Y, Fehr T, Giese N, Rosenbauer F, et al. Immunodeficiency and chronic myelogenous leukemia-like syndrome in mice with a targeted mutation of the ICSBP gene. Cell. 1996 Oct;87(2):307–317.

[31] Horie Y, Meguro A, Ota M, Kitaichi N, Katsuyama Y, Takemoto Y, et al. Association of TLR4 polymorphisms with Behcet's disease in a Korean population. Rheumatology (Oxford, England). 2009 Jun;48(6):638–642.

[32] Hou S, Yang P, Du L, Zhou H, Lin X, Liu X, et al. SUMO4 gene polymorphisms in Chinese Han patients with Behcet's disease. Clinical Immunology (Orlando, Fla). 2008 Oct;129(1):170–175.

[33] Hu J, Hou S, Zhu X, Fang J, Zhou Y, Liu Y, et al. Interleukin-10 gene polymorphisms are associated with Behcet's disease but not with Vogt-Koyanagi-Harada syndrome in the Chinese Han population. Molecular Vision. 2015;21:589–603.

[34] Jiang Y, Wang H, Yu H, Li L, Xu D, Hou S, et al. Two Genetic Variations in the IRF8 region are associated with Behçet's disease in Han Chinese. Scientific Reports. 2016 Jan;6:19651.

[35] Kamoun M, Ben Dhifallah I, Karray E, Zakraoui L, Hamzaoui K. Association of small ubiquitin-like modifier 4 (SUMO4) polymorphisms in a Tunisian population with Behçet's disease. Clinical and Experimental Rheumatology. 2010 Aug;28(4 Suppl 60):S45–49.

[36] Kang EH, Kim S, Park MY, Choi JY, Choi IA, Kim MJ, et al. Behçet's disease risk association fine-mapped on the IL23R-IL12RB2 intergenic region in Koreans. Arthritis Research & Therapy. 2017 Oct;19(1):227.

[37] Koumantaki Y, Stavropoulos C, Spyropoulou M, Messini H, Papademetropoulos M, Giziaki E, et al. HLA-B*5101 in Greek patients with Behçet's disease. Human Immunology. 1998 Apr;59(4):250–255.

[38] Lamhamedi-Cherradi SE, Zheng S, Hilliard BA, Xu L, Sun J, Alsheadat S, et al. Transcriptional regulation of type I diabetes by NF-kappa B. Journal of Immunology (Baltimore, Md: 1950). 2003 Nov;171(9):4886–4892.

[39] Lee EG, Boone DL, Chai S, Libby SL, Chien M, Lodolce JP, et al. Failure to regulate TNF-induced NF-kappaB and cell death responses in A20-deficient mice. Science (New York, NY). 2000 Sep;289(5488):2350–2354.

[40] Lennikov A, Alekberova Z, Goloeva R, Kitaichi N, Denisov L, Namba K, et al. Single center study on ethnic and clinical features of Behcet's disease in Moscow, Russia. Clinical Rheumatology. 2015 Feb;34(2):321–327. Available from: http://link.springer.com/10.1007/s10067-013-2442-9.

[41] Mathur AN, Chang HC, Zisoulis DG, Stritesky GL, Yu Q, O'Malley JT, et al. Stat3 and Stat4 direct development of IL-17-secreting Th cells. Journal of Immunology (Baltimore, Md: 1950). 2007 Apr;178(8):4901–4907.

[42] Meguro A, Ota M, Katsuyama Y, Oka A, Ohno S, Inoko H, et al. Association of the toll-like receptor 4 gene polymorphisms with Behcet's disease. Annals of the Rheumatic Diseases. 2008 May;67(5):725–727.

[43] Mizuki N, Ota M, Kimura M, Ohno S, Ando H, Katsuyama Y, et al. Triplet repeat polymorphism in the transmembrane region of the MICA gene: A strong association of six GCT repetitions with Behçet disease. Proceedings of the National Academy of Sciences. 1997 Feb;94(4):1298–1303. Available from: https://www.pnas.org/content/94/4/1298.

[44] Mizuki N, Ota M, Katsuyama Y, Yabuki K, Ando H, Yoshida M, et al. HLA class I genotyping including HLA-B*51 allele typing in the Iranian patients with Behçet's disease. Tissue Antigens. 2001 May;57(5):457–462.

[45] Mizuki N, Ota M, Katsuyama Y, Yabuki K, Ando H, Shiina T, et al. Sequencing-based typing of HLA-B*51 alleles and the significant association of HLA-B*5101 and -B*5108 with Behçet's disease in Greek patients. Tissue Antigens. 2002 Feb;59(2):118–121.

[46] Mizuki N, Ota M, Yabuki K, Katsuyama Y, Ando H, Palimeris GD, et al. Localization of the pathogenic gene of Behçet's disease by microsatellite analysis of three different populations. Investigative Ophthalmology & Visual Science. 2000 Nov;41(12):3702–3708.

[47] Mizuki N, Yabuki K, Ota M, Katsuyama Y, Ando H, Nomura E, et al. Analysis of microsatellite polymorphism around the HLA-B locus in Iranian patients with Behçet's disease: Mizuki et al : Microsatellite polymorphism of Iranian BD patients. Tissue Antigens. 2002 Nov;60(5):396–399. Available from: http://doi.wiley.com/10.1034/j.1399-0039.2002.600506.x.

[48] Mizuki N, Yabuki K, Ota M, Verity D, Katsuyama Y, Ando H, et al. Microsatellite mapping of a susceptible locus within the HLA region for Behçet's disease using Jordanian patients. Human Immunology. 2001 Feb;62(2):186–190. Available from: https://linkinghub.elsevier.com/retrieve/pii/S0198885900002469.

[49] Montes-Cano MA, Conde-Jaldón M, García-Lozano JR, Ortiz-Fernández L, Ortego-Centeno N, Castillo-Palma MJ, et al. HLA and non-HLA genes in Behçet's disease: a multicentric study in the Spanish population. Arthritis Research & Therapy. 2013 Oct;15(5):R145.

[50] Nerlov C. The C/EBP family of transcription factors: a paradigm for interaction between gene expression and proliferation control. Trends in Cell Biology. 2007 Jul;17(7):318–324.

[51] Nishiyama M, Takahashi M, Manaka Ki, Suzuki S, Saito M, Nakae K. Microsatellite polymorphisms of the MICA gene among Japanese patients with Behçet's disease. Canadian Journal of Ophthalmology. 2006 Apr;41(2):210–215. Available from: https://linkinghub.elsevier.com/retrieve/pii/S0008418206801183.

[52] Ortiz-Fernández L, Conde-Jaldón M, García-Lozano JR, Montes-Cano MA, Ortego-Centeno N, Castillo-Palma MJ, et al. GIMAP and Behçet disease: no association in the European population. Annals of the Rheumatic Diseases. 2014 Jul;73(7):1433–1434.

[53] Ouyang X, Zhang R, Yang J, Li Q, Qin L, Zhu C, et al. Transcription factor IRF8 directs a silencing programme for TH17 cell differentiation. Nature Communications. 2011;2:314.

[54] Park SH, Park KS, Seo YI, Min DJ, Kim WU, Kim TG, et al. Association of MICA Polymorphism with HLA-B51 and Disease Severity in Korean Patients with Behcet's Disease. Journal of Korean Medical Science. 2002 Jun;17(3):366–370. Available from: https://doi.org/10.3346/jkms.2002.17.3.366.

[55] Paul M, Klein T, Krause I, Molad Y, Narinsky R, Weinberger A. Allelic distribution of HLA-B*5 in HLA-B5-positive Israeli patients with Behçet's disease. Tissue Antigens. 2001 Sep;58(3):185–186.

[56] Peddle L, Zipperlen K, Melay B, Hefferton D, Rahman P. Association of SEEK1 polymorphisms in Crohn's disease. Human Immunology. 2004 Jul;65(7):706–709.

[57] Picco P, Porfirio B, Gattorno M, Buoncompagni A, Falcini F, Cusano R, et al. MICA gene polymorphisms in an Italian paediatric series of juvenile Behçet disease. International Journal of Molecular Medicine. 2002 Nov;10(5):575–578.

[58] Piga M, Paladini F, Lai S, Erre G, Passiu G, Carcassi C, et al. Genetics of Behçet's disease in Sardinia: two distinct extended HLA haplotypes harbour the B*51 allele in the normal population and in patients. Clinical and Experimental Rheumatology. 2012 Jun;30(3 Suppl 72):S51–56.

[59] Radouane A, Oudghiri M, Chakib A, Naya A, Belhouari A, El Malki A, et al. HLA-B*27 allele associated to Behçet's disease and to anterior uveitis in Moroccan patients. Annales De Biologie Clinique. 2011 Aug;69(4):419–424.

[60] Salvarani C, Boiardi L, Mantovani V, Olivieri I, Ciancio G, Cantini F, et al. Association of MICA alleles and HLA-B51 in Italian patients with Behçet's disease. The Journal of Rheumatology. 2001 Aug;28(8):1867–1870.

[61] Sarkar B, Kulharia M, Mantha AK. Understanding human thiol dioxygenase enzymes: structure to function, and biology to pathology. International Journal of Experimental Pathology. 2017 Apr;98(2):52–66.

[62] Stanford SM, Bottini N. PTPN22: the archetypal non-HLA autoimmunity gene. Nature Reviews Rheumatology. 2014 Oct;10(10):602–611.

[63] Strober W, Murray PJ, Kitani A, Watanabe T. Signalling pathways and molecular interactions of NOD1 and NOD2. Nature Reviews Immunology. 2006 Jan;6(1):9–20.

[64] Takeuchi M, Mizuki N, Meguro A, Ombrello MJ, Kirino Y, Satorius C, et al. Dense genotyping of immune-related loci implicates host responses to microbial exposure in Behçet's disease susceptibility. Nature Genetics. 2017 Mar;49(3):438–443.

[65] Touitou I, Magne X, Molinari N, Navarro A, Quellec AL, Picco P, et al. MEFV mutations in Behçet's disease. Human Mutation. 2000 Sep;16(3):271–272.

[66] Wallace GR, Verity DH, Delamaine LJ, Ohno S, Inoko H, Ota M, et al. MIC-A allele profiles and HLA class I associations in Behçet's disease. Immunogenetics. 1999 Jun;49(7-8):613–617. Available from: http://link.springer.com/10.1007/s002510050656.

[67] Watford WT, Hissong BD, Bream JH, Kanno Y, Muul L, O'Shea JJ. Signaling by IL-12 and IL-23 and the immunoregulatory roles of STAT4. Immunological Reviews. 2004 Dec;202:139–156.

[68] Wilkinson DG, Bhatt S, Chavrier P, Bravo R, Charnay P. Segment-specific expression of a zinc-finger gene in the developing nervous system of the mouse. Nature. 1989 Feb;337(6206):461–464.

[69] Yabuki K, Mizuki N, Ota M, Katsuyama Y, Palimeris G, Stavropoulos C, et al. Association of MICA gene and HLA-B*5101 with Behçet's disease in Greece. Investigative Ophthalmology & Visual Science. 1999 Aug;40(9):1921–1926.

[70] Yamazoe K, Meguro A, Takeuchi M, Shibuya E, Ohno S, Mizuki N. Comprehensive analysis of the association between UBAC2 polymorphisms and Behçet's disease in a Japanese population. Scientific Reports. 2017 Apr;7(1):742.

[71] Yu H, Zheng M, Zhang L, Li H, Zhu Y, Cheng L, et al. Identification of susceptibility SNPs in IL10 and IL23R-IL12RB2 for Behçet's disease in Han Chinese. The Journal of Allergy and Clinical Immunology. 2017 Feb;139(2):621–627.

[72] Zhang L, Yu H, Zheng M, Li H, Liu Y, Kijlstra A, et al. Association of ERAP1 Gene Polymorphisms With Behçet's Disease in Han Chinese. Investigative Ophthalmology & Visual Science. 2015 Sep;56(10):6029–6035.

[73] Zhou X, Wang J, Zou H, Ward MM, Weisman MH, Espitia MG, et al. MICA, a gene contributing strong susceptibility to ankylosing spondylitis. Annals of the Rheumatic Diseases. 2014 Aug;73(8):1552–1557.

[74] Köhler S, Doelken SC, Mungall CJ, Bauer S, Firth HV, Bailleul-Forestier I, et al. The Human Phenotype Ontology project: linking molecular biology and disease through phenotype data. Nucleic Acids Research. 2014 Jan;42(Database issue):D966–974.

[75] Birlea SA, Gowan K, Fain PR, Spritz RA. Genome-wide association study of generalized vitiligo in an isolated European founder population identifies SMOC2, in close proximity to IDDM8. The Journal of Investigative Dermatology. 2010 Mar;130(3):798–803.

[76] Cheong KA, Kim NH, Noh M, Lee AY. Three new single nucleotide polymorphisms identified by a genome-wide association study in Korean patients with vitiligo. Journal of Korean Medical Science. 2013 May;28(5):775–779.

[77] Jin Y, Birlea SA, Fain PR, Gowan K, Riccardi SL, Holland PJ, et al. Genome-wide analysis identifies a quantitative trait locus in the MHC class II region associated with generalized vitiligo age of onset. The Journal of Investigative Dermatology. 2011 Jun;131(6):1308–1312.

[78] Jin Y, Birlea SA, Fain PR, Ferrara TM, Ben S, Riccardi SL, et al. Genome-wide association analyses identify 13 new susceptibility loci for generalized vitiligo. Nature Genetics. 2012 May;44(6):676–680.

[79] Jin Y, Birlea SA, Fain PR, Gowan K, Riccardi SL, Holland PJ, et al. Variant of TYR and autoimmunity susceptibility loci in generalized vitiligo. The New England Journal of Medicine. 2010 May;362(18):1686–1697.

[80] Quan C, Ren YQ, Xiang LH, Sun LD, Xu AE, Gao XH, et al. Genome-wide association study for vitiligo identifies susceptibility loci at 6q27 and the MHC. Nature Genetics. 2010 Jul;42(7):614–618.

[81] Tang XF, Zhang Z, Hu DY, Xu AE, Zhou HS, Sun LD, et al. Association analyses identify three susceptibility Loci for vitiligo in the Chinese Han population. The Journal of Investigative Dermatology. 2013 Feb;133(2):403–410.

[82] Bracken S, Byrne G, Kelly J, Jackson J, Feighery C. Altered gene expression in highly purified enterocytes from patients with active coeliac disease. BMC genomics. 2008 Aug;9:377.

[83] Carr EJ, Niederer HA, Williams J, Harper L, Watts RA, Lyons PA, et al. Confirmation of the genetic association of CTLA4 and PTPN22 with ANCA-associated vasculitis. BMC medical genetics. 2009 Dec;10:121.

[84] Curley CR, Monsuur AJ, Wapenaar MC, Rioux JD, Wijmenga C. A functional candidate screen for coeliac disease genes. European journal of human genetics: EJHG. 2006 Nov;14(11):1215–1222.

[85] Diosdado B, Wapenaar MC, Franke L, Duran KJ, Goerres MJ, Hadithi M, et al. A microarray screen for novel candidate genes in coeliac disease pathogenesis. Gut. 2004 Jul;53(7):944–951.

[86] Dubois PCA, Trynka G, Franke L, Hunt KA, Romanos J, Curtotti A, et al. Multiple common variants for celiac disease influencing immune gene expression. Nature Genetics. 2010 Apr;42(4):295–302.

[87] Festen EAM, Goyette P, Green T, Boucher G, Beauchamp C, Trynka G, et al. A meta-analysis of genome-wide association scans identifies IL18RAP, PTPN2, TAGAP, and PUS10 as shared risk loci for Crohn's disease and celiac disease. PLoS genetics. 2011 Jan;7(1):e1001283.

[88] Garner C, Ahn R, Ding YC, Steele L, Stoven S, Green PH, et al. Genome-wide association study of celiac disease in North America confirms FRMD4B as new celiac locus. PloS One. 2014;9(7):e101428.

[89] Hunt KA, Zhernakova A, Turner G, Heap GAR, Franke L, Bruinenberg M, et al. Newly identified genetic risk variants for celiac disease related to the immune response. Nature Genetics. 2008 Apr;40(4):395–402.

[90] Li YR, Li J, Zhao SD, Bradfield JP, Mentch FD, Maggadottir SM, et al. Meta-analysis of shared genetic architecture across ten pediatric autoimmune diseases. Nature Medicine. 2015 Sep;21(9):1018–1027.

[91] Östensson M, Montén C, Bacelis J, Gudjonsdottir AH, Adamovic S, Ek J, et al. A possible mechanism behind autoimmune disorders discovered by genome-wide linkage and association analysis in celiac disease. PloS One. 2013;8(8):e70174.

[92] Tello-Ruiz MK, Curley C, DelMonte T, Giallourakis C, Kirby A, Miller K, et al. Haplotype-based association analysis of 56 functional candidate genes in the IBD6 locus on chromosome 19. European journal of human genetics: EJHG. 2006 Jun;14(6):780–790.

[93] van der Pouw Kraan TCTM, Zwiers A, Mulder CJ, Kraal G, Bouma G. Acute experimental colitis and human chronic inflammatory diseases share expression of inflammation-related genes with conserved Ets2 binding sites. Inflammatory Bowel Diseases. 2009 Feb;15(2):224–235.

[94] van Heel DA, Franke L, Hunt KA, Gwilliam R, Zhernakova A, Inouye M, et al. A genome-wide association study for celiac disease identifies risk variants in the region harboring IL2 and IL21. Nature Genetics. 2007 Jul;39(7):827–829.

[95] Zhernakova A, Stahl EA, Trynka G, Raychaudhuri S, Festen EA, Franke L, et al. Meta-analysis of genome-wide association studies in celiac disease and rheumatoid arthritis identifies fourteen non-HLA shared loci. PLoS genetics. 2011 Feb;7(2):e1002004.

[96] Alonso A, Domènech E, Julià A, Panés J, García-Sánchez V, Mateu PN, et al. Identification of risk loci for Crohn's disease phenotypes using a genome-wide association study. Gastroenterology. 2015 Apr;148(4):794–805.

[97] Barrett JC, Hansoul S, Nicolae DL, Cho JH, Duerr RH, Rioux JD, et al. Genome-wide association defines more than 30 distinct susceptibility loci for Crohn's disease. Nature Genetics. 2008 Aug;40(8):955–962.

[98] Dubinsky MC, Kugathasan S, Kwon S, Haritunians T, Wrobel I, Wahbeh G, et al. Multidimensional prognostic risk assessment identifies association between IL12B variation and surgery in Crohn's disease. Inflammatory Bowel Diseases. 2013 Jul;19(8):1662–1670.

[99] Ellinghaus D, Ellinghaus E, Nair RP, Stuart PE, Esko T, Metspalu A, et al. Combined analysis of genome-wide association studies for Crohn disease and psoriasis identifies seven shared susceptibility loci. American Journal of Human Genetics. 2012 Apr;90(4):636–647.

[100] Franke A, Fischer A, Nothnagel M, Becker C, Grabe N, Till A, et al. Genome-wide association analysis in sarcoidosis and Crohn's disease unravels a common susceptibility locus on 10p12.2. Gastroenterology. 2008 Oct;135(4):1207–1215.

[101] Franke A, Balschun T, Sina C, Ellinghaus D, Häsler R, Mayr G, et al. Genome-wide association study for ulcerative colitis identifies risk loci at 7q22 and 22q13 (IL17REL). Nature Genetics. 2010 Apr;42(4):292–294.

[102] Huang J, Ellinghaus D, Franke A, Howie B, Li Y. 1000 Genomes-based imputation identifies novel and refined associations for the Wellcome Trust Case Control Consortium phase 1 Data. European journal of human genetics: EJHG. 2012 Jul;20(7):801–805.

[103] Jostins L, Ripke S, Weersma RK, Duerr RH, McGovern DP, Hui KY, et al. Host-microbe interactions have shaped the genetic architecture of inflammatory bowel disease. Nature. 2012 Nov;491(7422):119–124.

[104] Jung ES, Cheon JH, Lee JH, Park SJ, Jang HW, Chung SH, et al. HLA-C*01 is a Risk Factor for Crohn's Disease. Inflammatory Bowel Diseases. 2016 Apr;22(4):796–806.

[105] Kenny EE, Pe'er I, Karban A, Ozelius L, Mitchell AA, Ng SM, et al. A genome-wide scan of Ashkenazi Jewish Crohn's disease suggests novel susceptibility loci. PLoS genetics. 2012;8(3):e1002559.

[106] Libioulle C, Louis E, Hansoul S, Sandor C, Farnir F, Franchimont D, et al. Novel Crohn disease locus identified by genome-wide association maps to a gene desert on 5p13.1 and modulates expression of PTGER4. PLoS genetics. 2007 Apr;3(4):e58.

[107] McGovern DPB, Jones MR, Taylor KD, Marciante K, Yan X, Dubinsky M, et al. Fucosyltransferase 2 (FUT2) non-secretor status is associated with Crohn's disease. Human Molecular Genetics. 2010 Sep;19(17):3468–3476.

[108] Okada Y, Yamazaki K, Umeno J, Takahashi A, Kumasaka N, Ashikawa K, et al. HLA-Cw*1202-B*5201-DRB1*1502 haplotype increases risk for ulcerative colitis but reduces risk for Crohn's disease. Gastroenterology. 2011 Sep;141(3):864–871.e1–5.

[109] Parkes M, Barrett JC, Prescott NJ, Tremelling M, Anderson CA, Fisher SA, et al. Sequence variants in the autophagy gene IRGM and multiple other replicating loci contribute to Crohn's disease susceptibility. Nature Genetics. 2007 Jul;39(7):830–832.

[110] Raelson JV, Little RD, Ruether A, Fournier H, Paquin B, Van Eerdewegh P, et al. Genome-wide association study for Crohn's disease in the Quebec Founder Population identifies multiple validated disease loci. Proceedings of the National Academy of Sciences of the United States of America. 2007 Sep;104(37):14747–14752.

[111] Rioux JD, Xavier RJ, Taylor KD, Silverberg MS, Goyette P, Huett A, et al. Genome-wide association study identifies new susceptibility loci for Crohn disease and implicates autophagy in disease pathogenesis. Nature Genetics. 2007 May;39(5):596–604.

[112] Yamazaki K, Umeno J, Takahashi A, Hirano A, Johnson TA, Kumasaka N, et al. A genome-wide association study identifies 2 susceptibility Loci for Crohn's disease in a Japanese population. Gastroenterology. 2013 Apr;144(4):781–788.

[113] Yang SK, Hong M, Zhao W, Jung Y, Baek J, Tayebi N, et al. Genome-wide association study of Crohn's disease in Koreans revealed three new susceptibility loci and common attributes of genetic susceptibility across ethnic populations. Gut. 2014 Jan;63(1):80–87.

[114] Baurecht H, Hotze M, Brand S, Büning C, Cormican P, Corvin A, et al. Genome-wide comparative analysis of atopic dermatitis and psoriasis gives insight into opposing genetic mechanisms. American Journal of Human Genetics. 2015 Jan;96(1):104–120.

[115] Capon F, Bijlmakers MJ, Wolf N, Quaranta M, Huffmeier U, Allen M, et al. Identification of ZNF313/RNF114 as a novel psoriasis susceptibility gene. Human Molecular Genetics. 2008 Jul;17(13):1938–1945.

[116] Ellinghaus E, Ellinghaus D, Stuart PE, Nair RP, Debrus S, Raelson JV, et al. Genome-wide association study identifies a psoriasis susceptibility locus at TRAF3IP2. Nature Genetics. 2010 Nov;42(11):991–995.

[117] Ellinghaus E, Stuart PE, Ellinghaus D, Nair RP, Debrus S, Raelson JV, et al. Genome-wide meta-analysis of psoriatic arthritis identifies susceptibility locus at REL. The Journal of Investigative Dermatology. 2012 Apr;132(4):1133–1140.

[118] Genetic Analysis of Psoriasis Consortium & the Wellcome Trust Case Control Consortium 2, Strange A, Capon F, Spencer CCA, Knight J, Weale ME, et al. A genome-wide association study identifies new psoriasis susceptibility loci and an interaction between HLA-C and ERAP1. Nature Genetics. 2010 Nov;42(11):985–990.

[119] Haider AS, Lowes MA, Suárez-Fariñas M, Zaba LC, Cardinale I, Khatcherian A, et al. Identification of cellular pathways of "type 1," Th17 T cells, and TNF- and inducible nitric oxide synthase-producing dendritic cells in autoimmune inflammation through pharmacogenomic study of cyclosporine A in psoriasis. Journal of Immunology (Baltimore, Md: 1950). 2008 Feb;180(3):1913–1920.

[120] Hüffmeier U, Uebe S, Ekici AB, Bowes J, Giardina E, Korendowych E, et al. Common variants at TRAF3IP2 are associated with susceptibility to psoriatic arthritis and psoriasis. Nature Genetics. 2010 Nov;42(11):996–999.

[121] Li YR, Li J, Zhao SD, Bradfield JP, Mentch FD, Maggadottir SM, et al. Meta-analysis of shared genetic architecture across ten pediatric autoimmune diseases. Nature Medicine. 2015 Sep;21(9):1018–1027.

[122] Liu Y, Helms C, Liao W, Zaba LC, Duan S, Gardner J, et al. A genome-wide association study of psoriasis and psoriatic arthritis identifies new disease loci. PLoS genetics. 2008 Mar;4(3):e1000041.

[123] Nair RP, Duffin KC, Helms C, Ding J, Stuart PE, Goldgar D, et al. Genome-wide scan reveals association of psoriasis with IL-23 and NF-kappaB pathways. Nature Genetics. 2009 Feb;41(2):199–204.

[124] Stuart PE, Nair RP, Tsoi LC, Tejasvi T, Das S, Kang HM, et al. Genome-wide Association Analysis of Psoriatic Arthritis and Cutaneous Psoriasis Reveals Differences in Their Genetic Architecture. American Journal of Human Genetics. 2015 Dec;97(6):816–836.

[125] Stuart PE, Nair RP, Ellinghaus E, Ding J, Tejasvi T, Gudjonsson JE, et al. Genome-wide association analysis identifies three psoriasis susceptibility loci. Nature Genetics. 2010 Nov;42(11):1000–1004.

[126] Tsoi LC, Spain SL, Ellinghaus E, Stuart PE, Capon F, Knight J, et al. Enhanced meta-analysis and replication studies identify five new psoriasis susceptibility loci. Nature Communications. 2015 May;6:7001.

[127] Yin X, Low HQ, Wang L, Li Y, Ellinghaus E, Han J, et al. Genome-wide meta-analysis identifies multiple novel associations and ethnic heterogeneity of psoriasis susceptibility. Nature Communications. 2015 Apr;6:6916.

[128] Zhang XJ, Huang W, Yang S, Sun LD, Zhang FY, Zhu QX, et al. Psoriasis genome-wide association study identifies susceptibility variants within LCE gene cluster at 1q21. Nature Genetics. 2009 Feb;41(2):205–210.

[129] Awata T, Yamashita H, Kurihara S, Morita-Ohkubo T, Miyashita Y, Katayama S, et al. A genome-wide association study for diabetic retinopathy in a Japanese population: potential association with a long intergenic non-coding RNA. PloS One. 2014;9(11):e111715.

[130] Blackman SM, Commander CW, Watson C, Arcara KM, Strug LJ, Stonebraker JR, et al. Genetic modifiers of cystic fibrosis-related diabetes. Diabetes. 2013 Oct;62(10):3627–3635.

[131] Burdon KP, Fogarty RD, Shen W, Abhary S, Kaidonis G, Appukuttan B, et al. Genome-wide association study for sight-threatening diabetic retinopathy reveals association with genetic variation near the GRB2 gene. Diabetologia. 2015 Oct;58(10):2288–2297.

[132] Germain M, Pezzolesi MG, Sandholm N, McKnight AJ, Susztak K, Lajer M, et al. SORBS1 gene, a new candidate for diabetic nephropathy: results from a multi-stage genome-wide association study in patients with type 1 diabetes. Diabetologia. 2015 Mar;58(3):543–548.

[133] Grassi MA, Tikhomirov A, Ramalingam S, Below JE, Cox NJ, Nicolae DL. Genome-wide meta-analysis for severe diabetic retinopathy. Human Molecular Genetics. 2011 Jun;20(12):2472–2481.

[134] Hanson RL, Muller YL, Kobes S, Guo T, Bian L, Ossowski V, et al. A genome-wide association study in American Indians implicates DNER as a susceptibility locus for type 2 diabetes. Diabetes. 2014 Jan;63(1):369–376.

[135] Huang YC, Lin JM, Lin HJ, Chen CC, Chen SY, Tsai CH, et al. Genome-wide association study of diabetic retinopathy in a Taiwanese population. Ophthalmology. 2011 Apr;118(4):642–648.

[136] Imamura M, Maeda S, Yamauchi T, Hara K, Yasuda K, Morizono T, et al. A single-nucleotide polymorphism in ANK1 is associated with susceptibility to type 2 diabetes in Japanese populations. Human Molecular Genetics. 2012 Jul;21(13):3042–3049.

[137] Iyengar SK, Sedor JR, Freedman BI, Kao WHL, Kretzler M, Keller BJ, et al. Genome-Wide Association and Trans-ethnic Meta-Analysis for Advanced Diabetic Kidney Disease: Family Investigation of Nephropathy and Diabetes (FIND). PLoS genetics. 2015 Aug;11(8):e1005352.

[138] Kwak SH, Kim SH, Cho YM, Go MJ, Cho YS, Choi SH, et al. A genome-wide association study of gestational diabetes mellitus in Korean women. Diabetes. 2012 Feb;61(2):531–541.

[139] Li H, Gan W, Lu L, Dong X, Han X, Hu C, et al. A genome-wide association study identifies GRK5 and RASGRP1 as type 2 diabetes loci in Chinese Hans. Diabetes. 2013 Jan;62(1):291–298.

[140] Maeda S, Osawa N, Hayashi T, Tsukada S, Kobayashi M, Kikkawa R. Genetic variations associated with diabetic nephropathy and type II diabetes in a Japanese population. Kidney International Supplement. 2007 Aug;(106):S43–48.

[141] McDonough CW, Palmer ND, Hicks PJ, Roh BH, An SS, Cooke JN, et al. A genome-wide association study for diabetic nephropathy genes in African Americans. Kidney International. 2011 Mar;79(5):563–572.

[142] Meng W, Deshmukh HA, Donnelly LA, Wellcome Trust Case Control Consortium 2 (WTCCC2), Surrogate markers for Micro- and Macro-vascular hard endpoints for Innovative diabetes Tools (SUMMIT) study group, Torrance N, et al. A Genome-wide Association Study Provides Evidence of Sex-specific Involvement of Chr1p35.1 (ZSCAN20-TLR12P) and Chr8p23.1 (HMGB1P46) With Diabetic Neuropathic Pain. EBioMedicine. 2015 Oct;2(10):1386–1393.

[143] Perry JRB, Voight BF, Yengo L, Amin N, Dupuis J, Ganser M, et al. Stratifying type 2 diabetes cases by BMI identifies genetic risk variants in LAMA1 and enrichment for risk variants in lean compared to obese cases. PLoS genetics. 2012 May;8(5):e1002741.

[144] Saxena R, Saleheen D, Been LF, Garavito ML, Braun T, Bjonnes A, et al. Genome-wide association study identifies a novel locus contributing to type 2 diabetes susceptibility in Sikhs of Punjabi origin from India. Diabetes. 2013 May;62(5):1746–1755.

[145] SIGMA Type 2 Diabetes Consortium, Williams AL, Jacobs SBR, Moreno-Macías H, Huerta-Chagoya A, Churchhouse C, et al. Sequence variants in SLC16A11 are a common risk factor for type 2 diabetes in Mexico. Nature. 2014 Feb;506(7486):97–101.

[146] Tabassum R, Chauhan G, Dwivedi OP, Mahajan A, Jaiswal A, Kaur I, et al. Genome-wide association study for type 2 diabetes in Indians identifies a new susceptibility locus at 2q21. Diabetes. 2013 Mar;62(3):977–986.

[147] Takeuchi F, Serizawa M, Yamamoto K, Fujisawa T, Nakashima E, Ohnaka K, et al. Confirmation of multiple risk Loci and genetic impacts by a genome-wide association study of type 2 diabetes in the Japanese population. Diabetes. 2009 Jul;58(7):1690–1699.

[148] Timpson NJ, Lindgren CM, Weedon MN, Randall J, Ouwehand WH, Strachan DP, et al. Adiposity-related heterogeneity in patterns of type 2 diabetes susceptibility observed in genome-wide association data. Diabetes. 2009 Feb;58(2):505–510.

[149] Fischer A, Schmid B, Ellinghaus D, Nothnagel M, Gaede KI, Schürmann M, et al. A novel sarcoidosis risk locus for Europeans on chromosome 11q13.1. American Journal of Respiratory and Critical Care Medicine. 2012 Nov;186(9):877–885.

[150] Hofmann S, Fischer A, Nothnagel M, Jacobs G, Schmid B, Wittig M, et al. Genome-wide association analysis reveals 12q13.3-q14.1 as new risk locus for sarcoidosis. The European Respiratory Journal. 2013 Apr;41(4):888–900.

[151] Miller FW, Chen W, O'Hanlon TP, Cooper RG, Vencovsky J, Rider LG, et al. Genome-wide association study identifies HLA 8.1 ancestral haplotype alleles as major genetic risk factors for myositis phenotypes. Genes and Immunity. 2015 Oct;16(7):470–480.

[152] Allanore Y, Saad M, Dieudé P, Avouac J, Distler JHW, Amouyel P, et al. Genome-wide scan identifies TNIP1, PSORS1C1, and RHOB as novel risk loci for systemic sclerosis. PLoS genetics. 2011 Jul;7(7):e1002091.

[153] Gorlova O, Martin JE, Rueda B, Koeleman BPC, Ying J, Teruel M, et al. Identification of novel genetic markers associated with clinical phenotypes of systemic sclerosis through a genome-wide association strategy. PLoS genetics. 2011 Jul;7(7):e1002178.

[154] Martin JE, Assassi S, Diaz-Gallo LM, Broen JC, Simeon CP, Castellvi I, et al. A systemic sclerosis and systemic lupus erythematosus pan-meta-GWAS reveals new shared susceptibility loci. Human Molecular Genetics. 2013 Oct;22(19):4021–4029.

[155] Radstake TRDJ, Gorlova O, Rueda B, Martin JE, Alizadeh BZ, Palomino-Morales R, et al. Genome-wide association study of systemic sclerosis identifies CD247 as a new susceptibility locus. Nature Genetics. 2010 May;42(5):426–429.

[156] Petukhova L, Duvic M, Hordinsky M, Norris D, Price V, Shimomura Y, et al. Genome-wide association study in alopecia areata implicates both innate and adaptive immunity. Nature. 2010 Jul;466(7302):113–117.

[157] Schormair B, Kemlink D, Roeske D, Eckstein G, Xiong L, Lichtner P, et al. PTPRD (protein tyrosine phosphatase receptor type delta) is associated with restless legs syndrome. Nature Genetics. 2008 Aug;40(8):946–948.

[158] Stefansson H, Rye DB, Hicks A, Petursson H, Ingason A, Thorgeirsson TE, et al. A genetic risk factor for periodic limb movements in sleep. The New England Journal of Medicine. 2007 Aug;357(7):639–647.

[159] Winkelmann J, Czamara D, Schormair B, Knauf F, Schulte EC, Trenkwalder C, et al. Genome-wide association study identifies novel restless legs syndrome susceptibility loci on 2p14 and 16q12.1. PLoS genetics. 2011 Jul;7(7):e1002171.

[160] Winkelmann J, Schormair B, Lichtner P, Ripke S, Xiong L, Jalilzadeh S, et al. Genome-wide association study of restless legs syndrome identifies common variants in three genomic regions. Nature Genetics. 2007 Aug;39(8):1000–1006.

[161] Clancy RM, Marion MC, Kaufman KM, Ramos PS, Adler A, International Consortium on Systemic Lupus Erythematosus Genetics, et al. Identification of candidate loci at 6p21 and 21q22 in a genome-wide association study of cardiac manifestations of neonatal lupus. Arthritis and Rheumatism. 2010 Nov;62(11):3415–3424.

[162] Ramos-Casals M, Brito-Zeron P, Siso-Almirall A, Bosch X. Primary Sjogren syndrome. BMJ. 2012 Jun;344(jun14 1):e3821–e3821. Available from: `http://www.bmj.com/cgi/doi/10.1136/bmj.e3821`.

[163] Song IW, Chen HC, Lin YF, Yang JH, Chang CC, Chou CT, et al. Identification of susceptibility gene associated with female primary Sjögren's syndrome in Han Chinese by genome-wide association study. Human Genetics. 2016 Nov;135(11):1287–1294.

[164] Medici M, Porcu E, Pistis G, Teumer A, Brown SJ, Jensen RA, et al. Identification of novel genetic Loci associated with thyroid peroxidase antibodies and clinical thyroid disease. PLoS genetics. 2014 Feb;10(2):e1004123.

[165] Oryoji D, Ueda S, Yamamoto K, Yoshimura Noh J, Okamura K, Noda M, et al. Identification of a Hashimoto thyroiditis susceptibility locus via a genome-wide comparison with Graves' disease. The Journal of Clinical Endocrinology and Metabolism. 2015 Feb;100(2):E319–324.

[166] Duncan EL, Danoy P, Kemp JP, Leo PJ, McCloskey E, Nicholson GC, et al. Genome-wide association study using extreme truncate selection identifies novel genes affecting bone mineral density and fracture risk. PLoS genetics. 2011 Apr;7(4):e1001372.

[167] Australo-Anglo-American Spondyloarthritis Consortium (TASC), Reveille JD, Sims AM, Danoy P, Evans DM, Leo P, et al. Genome-wide association study of ankylosing spondylitis identifies non-MHC susceptibility loci. Nature Genetics. 2010 Feb;42(2):123–127.

[168] Evans DM, Spencer CCA, Pointon JJ, Su Z, Harvey D, Kochan G, et al. Interaction between ERAP1 and HLA-B27 in ankylosing spondylitis implicates peptide handling in the mechanism for HLA-B27 in disease susceptibility. Nature Genetics. 2011 Jul;43(8):761–767.

[169] Lin Z, Bei JX, Shen M, Li Q, Liao Z, Zhang Y, et al. A genome-wide association study in Han Chinese identifies new susceptibility loci for ankylosing spondylitis. Nature Genetics. 2011 Dec;44(1):73–77.

[170] Deng X, Sabino EC, Cunha-Neto E, Ribeiro AL, Ianni B, Mady C, et al. Genome wide association study (GWAS) of Chagas cardiomyopathy in Trypanosoma cruzi seropositive subjects. PloS One. 2013;8(11):e79629.

[171] Andlauer TFM, Buck D, Antony G, Bayas A, Bechmann L, Berthele A, et al. Novel multiple sclerosis susceptibility loci implicated in epigenetic regulation. Science Advances. 2016;2(6):e1501678.

[172] Aulchenko YS, Hoppenbrouwers IA, Ramagopalan SV, Broer L, Jafari N, Hillert J, et al. Genetic variation in the KIF1B locus influences susceptibility to multiple sclerosis. Nature Genetics. 2008 Dec;40(12):1402–1403.

[173] Australia and New Zealand Multiple Sclerosis Genetics Consortium (ANZgene). Genome-wide association study identifies new multiple sclerosis susceptibility loci on chromosomes 12 and 20. Nature Genetics. 2009 Jul;41(7):824–828.

[174] Baranzini SE, Srinivasan R, Khankhanian P, Okuda DT, Nelson SJ, Matthews PM, et al. Genetic variation influences glutamate concentrations in brains of patients with multiple sclerosis. Brain: A Journal of Neurology. 2010 Sep;133(9):2603–2611.

[175] Baranzini SE, Wang J, Gibson RA, Galwey N, Naegelin Y, Barkhof F, et al. Genome-wide association analysis of susceptibility and clinical phenotype in multiple sclerosis. Human Molecular Genetics. 2009 Feb;18(4):767–778.

[176] Brynedal B, Wojcik J, Esposito F, Debailleul V, Yaouanq J, Martinelli-Boneschi F, et al. MGAT5 alters the severity of multiple sclerosis. Journal of Neuroimmunology. 2010 Mar;220(1-2):120–124.

[177] Comabella M, Craig DW, Camiña-Tato M, Morcillo C, Lopez C, Navarro A, et al. Identification of a novel risk locus for multiple sclerosis at 13q31.3 by a pooled genome-wide scan of 500,000 single nucleotide polymorphisms. PloS One. 2008;3(10):e3490.

[178] De Jager PL, Jia X, Wang J, de Bakker PIW, Ottoboni L, Aggarwal NT, et al. Meta-analysis of genome scans and replication identify CD6, IRF8 and TNFRSF1A as new multiple sclerosis susceptibility loci. Nature Genetics. 2009 Jul;41(7):776–782.

[179] Goris A, Pauwels I, Gustavsen MW, van Son B, Hilven K, Bos SD, et al. Genetic variants are major determinants of CSF antibody levels in multiple sclerosis. Brain: A Journal of Neurology. 2015 Mar;138(Pt 3):632–643.

[180] Goris A, van Setten J, Diekstra F, Ripke S, Patsopoulos NA, Sawcer SJ, et al. No evidence for shared genetic basis of common variants in multiple sclerosis and amyotrophic lateral sclerosis. Human Molecular Genetics. 2014 Apr;23(7):1916–1922.

[181] Gourraud PA, Sdika M, Khankhanian P, Henry RG, Beheshtian A, Matthews PM, et al. A genome-wide association study of brain lesion distribution in multiple sclerosis. Brain: A Journal of Neurology. 2013 Apr;136(Pt 4):1012–1024.

[182] International Multiple Sclerosis Genetics Consortium, Wellcome Trust Case Control Consortium 2, Sawcer S, Hellenthal G, Pirinen M, Spencer CCA, et al. Genetic risk and a primary role for cell-mediated immune mechanisms in multiple sclerosis. Nature. 2011 Aug;476(7359):214–219.

[183] International Multiple Sclerosis Genetics Consortium. Genome-wide association study of severity in multiple sclerosis. Genes and Immunity. 2011 Dec;12(8):615–625.

[184] International Multiple Sclerosis Genetics Consortium, Hafler DA, Compston A, Sawcer S, Lander ES, Daly MJ, et al. Risk alleles for multiple sclerosis identified by a genomewide study. The New England Journal of Medicine. 2007 Aug;357(9):851–862.

[185] Jakkula E, Leppä V, Sulonen AM, Varilo T, Kallio S, Kemppinen A, et al. Genome-wide association study in a high-risk isolate for multiple sclerosis reveals associated variants in STAT3 gene. American Journal of Human Genetics. 2010 Feb;86(2):285–291.

[186] Leone MA, Barizzone N, Esposito F, Lucenti A, Harbo HF, Goris A, et al. Association of genetic markers with CSF oligoclonal bands in multiple sclerosis patients. PloS One. 2013;8(6):e64408.

[187] Martinelli-Boneschi F, Esposito F, Brambilla P, Lindström E, Lavorgna G, Stankovich J, et al. A genome-wide association study in progressive multiple sclerosis. Multiple Sclerosis (Houndmills, Basingstoke, England). 2012 Oct;18(10):1384–1394.

[188] Matesanz F, González-Pérez A, Lucas M, Sanna S, Gayán J, Urcelay E, et al. Genome-wide association study of multiple sclerosis confirms a novel locus at 5p13.1. PloS One. 2012;7(5):e36140.

[189] Mero IL, Gustavsen MW, Sæther HS, Flåm ST, Berg-Hansen P, Søndergaard HB, et al. Oligoclonal band status in Scandinavian multiple sclerosis patients is associated with specific genetic risk alleles. PloS One. 2013;8(3):e58352.

[190] Nischwitz S, Cepok S, Kroner A, Wolf C, Knop M, Müller-Sarnowski F, et al. Evidence for VAV2 and ZNF433 as susceptibility genes for multiple sclerosis. Journal of Neuroimmunology. 2010 Oct;227(1-2):162–166.

[191] Patsopoulos NA, Bayer Pharma MS Genetics Working Group, Steering Committees of Studies Evaluating IFN-1b and a CCR1-Antagonist, ANZgene Consortium, GeneMSA, International Multiple Sclerosis Genetics Consortium, et al. Genome-wide meta-analysis identifies novel multiple sclerosis susceptibility loci. Annals of Neurology. 2011 Dec;70(6):897–912.

[192] Sanna S, Pitzalis M, Zoledziewska M, Zara I, Sidore C, Murru R, et al. Variants within the immunoregulatory CBLB gene are associated with multiple sclerosis. Nature Genetics. 2010 Jun;42(6):495–497.

[193] Wang JH, Pappas D, De Jager PL, Pelletier D, de Bakker PI, Kappos L, et al. Modeling the cumulative genetic risk for multiple sclerosis from genome-wide association data. Genome Medicine. 2011 Jan;3(1):3.

[194] Zhou Y, Zhu G, Charlesworth JC, Simpson S, Rubicz R, Göring HH, et al. Genetic loci for Epstein-Barr virus nuclear antigen-1 are associated with risk of multiple sclerosis. Multiple Sclerosis (Houndmills, Basingstoke, England). 2016;22(13):1655–1664.

[195] Adachi S, Tajima A, Quan J, Haino K, Yoshihara K, Masuzaki H, et al. Meta-analysis of genome-wide association scans for genetic susceptibility to endometriosis in Japanese population. Journal of Human Genetics. 2010 Dec;55(12):816–821.

[196] Albertsen HM, Chettier R, Farrington P, Ward K. Genome-wide association study link novel loci to endometriosis. PloS One. 2013;8(3):e58257.

[197] Nyholt DR, Low SK, Anderson CA, Painter JN, Uno S, Morris AP, et al. Genome-wide association meta-analysis identifies new endometriosis risk loci. Nature Genetics. 2012 Dec;44(12):1355–1359.

[198] Painter JN, Anderson CA, Nyholt DR, Macgregor S, Lin J, Lee SH, et al. Genome-wide association study identifies a locus at 7p15.2 associated with endometriosis. Nature Genetics. 2011 Jan;43(1):51–54.

[199] Uno S, Zembutsu H, Hirasawa A, Takahashi A, Kubo M, Akahane T, et al. A genome-wide association study identifies genetic variants in the CDKN2BAS locus associated with endometriosis in Japanese. Nature Genetics. 2010 Aug;42(8):707–710.

[200] Wang W, Li Y, Li S, Wu Z, Yuan M, Wang T, et al. Pooling-Based Genome-Wide Association Study Identifies Risk Loci in the Pathogenesis of Ovarian Endometrioma in Chinese Han Women. Reproductive Sciences (Thousand Oaks, Calif). 2017;24(3):400–406.

[201] Anderson CA, Boucher G, Lees CW, Franke A, D'Amato M, Taylor KD, et al. Meta-analysis identifies 29 additional ulcerative colitis risk loci, increasing the number of confirmed associations to 47. Nature Genetics. 2011 Mar;43(3):246–252.

[202] Asano K, Matsushita T, Umeno J, Hosono N, Takahashi A, Kawaguchi T, et al. A genome-wide association study identifies three new susceptibility loci for ulcerative colitis in the Japanese population. Nature Genetics. 2009 Dec;41(12):1325–1329.

[203] Burczynski ME, Peterson RL, Twine NC, Zuberek KA, Brodeur BJ, Casciotti L, et al. Molecular classification of Crohn's disease and ulcerative colitis patients using transcriptional profiles in peripheral blood mononuclear cells. The Journal of molecular diagnostics: JMD. 2006 Feb;8(1):51–61.

[204] Ellinghaus D, Folseraas T, Holm K, Ellinghaus E, Melum E, Balschun T, et al. Genome-wide association analysis in primary sclerosing cholangitis and ulcerative colitis identifies risk loci at GPR35 and TCF4. Hepatology (Baltimore, Md). 2013 Sep;58(3):1074–1083.

[205] Flach CF, Eriksson A, Jennische E, Lange S, Gunnerek C, Lönnroth I. Detection of elafin as a candidate biomarker for ulcerative colitis by whole-genome microarray screening. Inflammatory Bowel Diseases. 2006 Sep;12(9):837–842.

[206] Franke A, Balschun T, Karlsen TH, Sventoraityte J, Nikolaus S, Mayr G, et al. Sequence variants in IL10, ARPC2 and multiple other loci contribute to ulcerative colitis susceptibility. Nature Genetics. 2008 Nov;40(11):1319–1323.

[207] Haritunians T, Taylor KD, Targan SR, Dubinsky M, Ippoliti A, Kwon S, et al. Genetic predictors of medically refractory ulcerative colitis. Inflammatory Bowel Diseases. 2010 Nov;16(11):1830–1840.

[208] Julià A, Domènech E, Chaparro M, García-Sánchez V, Gomollón F, Panés J, et al. A genome-wide association study identifies a novel locus at 6q22.1 associated with ulcerative colitis. Human Molecular Genetics. 2014 Dec;23(25):6927–6934.

[209] Juyal G, Negi S, Sood A, Gupta A, Prasad P, Senapati S, et al. Genome-wide association scan in north Indians reveals three novel HLA-independent risk loci for ulcerative colitis. Gut. 2015 Apr;64(4):571–579.

[210] Liu JZ, van Sommeren S, Huang H, Ng SC, Alberts R, Takahashi A, et al. Association analyses identify 38 susceptibility loci for inflammatory bowel disease and highlight shared genetic risk across populations. Nature Genetics. 2015 Sep;47(9):979–986.

[211] McGovern DPB, Gardet A, Törkvist L, Goyette P, Essers J, Taylor KD, et al. Genome-wide association identifies multiple ulcerative colitis susceptibility loci. Nature Genetics. 2010 Apr;42(4):332–337.

[212] Okahara S, Arimura Y, Yabana T, Kobayashi K, Gotoh A, Motoya S, et al. Inflammatory gene signature in ulcerative colitis with cDNA macroarray analysis. Alimentary Pharmacology & Therapeutics. 2005 May;21(9):1091–1097.

[213] Silverberg MS, Cho JH, Rioux JD, McGovern DPB, Wu J, Annese V, et al. Ulcerative colitis-risk loci on chromosomes 1p36 and 12q15 found by genome-wide association study. Nature Genetics. 2009 Feb;41(2):216–220.

[214] UK IBD Genetics Consortium, Barrett JC, Lee JC, Lees CW, Prescott NJ, Anderson CA, et al. Genome-wide association study of ulcerative colitis identifies three new susceptibility loci, including the HNF4A region. Nature Genetics. 2009 Dec;41(12):1330–1334.

[215] Waller S, Tremelling M, Bredin F, Godfrey L, Howson J, Parkes M. Evidence for association of OCTN genes and IBD5 with ulcerative colitis. Gut. 2006 Jun;55(6):809–814.

[216] Watanabe T, Kobunai T, Toda E, Kanazawa T, Kazama Y, Tanaka J, et al. Gene expression signature and the prediction of ulcerative colitis-associated colorectal cancer by DNA microarray. Clinical Cancer Research: An Official Journal of the American Association for Cancer Research. 2007 Jan;13(2 Pt 1):415–420.

[217] Yang SK, Hong M, Zhao W, Jung Y, Tayebi N, Ye BD, et al. Genome-wide association study of ulcerative colitis in Koreans suggests extensive overlapping of genetic susceptibility with Caucasians. Inflammatory Bowel Diseases. 2013 Apr;19(5):954–966.

[218] Zahn A, Moehle C, Langmann T, Ehehalt R, Autschbach F, Stremmel W, et al. Aquaporin-8 expression is reduced in ileum and induced in colon of patients with ulcerative colitis. World Journal of Gastroenterology. 2007 Mar;13(11):1687–1695.

[219] Milton JN, Sebastiani P, Solovieff N, Hartley SW, Bhatnagar P, Arking DE, et al. A genome-wide association study of total bilirubin and cholelithiasis risk in sickle cell anemia. PloS One. 2012;7(4):e34741.

[220] Alarcón-Riquelme ME, Ziegler JT, Molineros J, Howard TD, Moreno-Estrada A, Sánchez-Rodríguez E, et al. Genome-Wide Association Study in an Amerindian Ancestry Population Reveals Novel Systemic Lupus Erythematosus Risk Loci and the Role of European Admixture. Arthritis & Rheumatology (Hoboken, NJ). 2016 Apr;68(4):932–943.

[221] Armstrong DL, Zidovetzki R, Alarcón-Riquelme ME, Tsao BP, Criswell LA, Kimberly RP, et al. GWAS identifies novel SLE susceptibility genes and explains the association of the HLA region. Genes and Immunity. 2014 Sep;15(6):347–354.

[222] Bentham J, Morris DL, Graham DSC, Pinder CL, Tombleson P, Behrens TW, et al. Genetic association analyses implicate aberrant regulation of innate and adaptive immunity genes in the pathogenesis of systemic lupus erythematosus. Nature Genetics. 2015 Dec;47(12):1457–1464.

[223] Chung SA, Taylor KE, Graham RR, Nititham J, Lee AT, Ortmann WA, et al. Differential genetic associations for systemic lupus erythematosus based on anti-dsDNA autoantibody production. PLoS genetics. 2011 Mar;7(3):e1001323.

[224] Demirci FY, Wang X, Kelly JA, Morris DL, Barmada MM, Feingold E, et al. Identification of a New Susceptibility Locus for Systemic Lupus Erythematosus on Chromosome 12 in Individuals of European Ancestry. Arthritis & Rheumatology (Hoboken, NJ). 2016 Jan;68(1):174–183.

[225] Graham RR, Cotsapas C, Davies L, Hackett R, Lessard CJ, Leon JM, et al. Genetic variants near TNFAIP3 on 6q23 are associated with systemic lupus erythematosus. Nature Genetics. 2008 Sep;40(9):1059–1061.

[226] Han JW, Zheng HF, Cui Y, Sun LD, Ye DQ, Hu Z, et al. Genome-wide association study in a Chinese Han population identifies nine new susceptibility loci for systemic lupus erythematosus. Nature Genetics. 2009 Nov;41(11):1234–1237.

[227] Hom G, Graham RR, Modrek B, Taylor KE, Ortmann W, Garnier S, et al. Association of systemic lupus erythematosus with C8orf13-BLK and ITGAM-ITGAX. The New England Journal of Medicine. 2008 Feb;358(9):900–909.

[228] International Consortium for Systemic Lupus Erythematosus Genetics (SLEGEN), Harley JB, Alarcón-Riquelme ME, Criswell LA, Jacob CO, Kimberly RP, et al. Genome-wide association scan in women with systemic lupus erythematosus identifies susceptibility variants in ITGAM, PXK, KIAA1542 and other loci. Nature Genetics. 2008 Feb;40(2):204–210.

[229] Kariuki SN, Ghodke-Puranik Y, Dorschner JM, Chrabot BS, Kelly JA, Tsao BP, et al. Genetic analysis of the pathogenic molecular sub-phenotype interferon-alpha identifies multiple novel loci involved in systemic lupus erythematosus. Genes and Immunity. 2015 Feb;16(1):15–23.

[230] Kim K, Bang SY, Joo YB, Kim T, Lee HS, Kang C, et al. Response to Intravenous Cyclophosphamide Treatment for Lupus Nephritis Associated with Polymorphisms in the FCGR2B-FCRLA Locus. The Journal of Rheumatology. 2016;43(6):1045–1049.

[231] Kozyrev SV, Abelson AK, Wojcik J, Zaghlool A, Linga Reddy MVP, Sanchez E, et al. Functional variants in the B-cell gene BANK1 are associated with systemic lupus erythematosus. Nature Genetics. 2008 Feb;40(2):211–216.

[232] Lee YH, Bae SC, Choi SJ, Ji JD, Song GG. Genome-wide pathway analysis of genome-wide association studies on systemic lupus erythematosus and rheumatoid arthritis. Molecular Biology Reports. 2012 Dec;39(12):10627–10635.

[233] Lessard CJ, Sajuthi S, Zhao J, Kim K, Ice JA, Li H, et al. Identification of a Systemic Lupus Erythematosus Risk Locus Spanning ATG16L2, FCHSD2, and P2RY2 in Koreans. Arthritis & Rheumatology (Hoboken, NJ). 2016;68(5):1197–1209.

[234] Márquez A, Vidal-Bralo L, Rodríguez-Rodríguez L, González-Gay MA, Balsa A, González-Álvaro I, et al. A combined large-scale meta-analysis identifies COG6 as a novel shared risk locus for rheumatoid arthritis and systemic lupus erythematosus. Annals of the Rheumatic Diseases. 2017 Jan;76(1):286–294.

[235] Morris DL, Sheng Y, Zhang Y, Wang YF, Zhu Z, Tombleson P, et al. Genome-wide association meta-analysis in Chinese and European individuals identifies ten new loci associated with systemic lupus erythematosus. Nature Genetics. 2016;48(8):940–946.

[236] Okada Y, Shimane K, Kochi Y, Tahira T, Suzuki A, Higasa K, et al. A genome-wide association study identified AFF1 as a susceptibility locus for systemic lupus eyrthematosus in Japanese. PLoS genetics. 2012 Jan;8(1):e1002455.

[237] Yang J, Yang W, Hirankarn N, Ye DQ, Zhang Y, Pan HF, et al. ELF1 is associated with systemic lupus erythematosus in Asian populations. Human Molecular Genetics. 2011 Feb;20(3):601–607.

[238] Yang W, Shen N, Ye DQ, Liu Q, Zhang Y, Qian XX, et al. Genome-wide association study in Asian populations identifies variants in ETS1 and WDFY4 associated with systemic lupus erythematosus. PLoS genetics. 2010 Feb;6(2):e1000841.

[239] Zhang Y, Yang J, Zhang J, Sun L, Hirankarn N, Pan HF, et al. Genome-wide search followed by replication reveals genetic interaction of CD80 and ALOX5AP associated with systemic lupus erythematosus in Asian populations. Annals of the Rheumatic Diseases. 2016 May;75(5):891–898.

[240] Cordell HJ, Han Y, Mells GF, Li Y, Hirschfield GM, Greene CS, et al. International genome-wide meta-analysis identifies new primary biliary cirrhosis risk loci and targetable pathogenic pathways. Nature Communications. 2015 Sep;6:8019.

[241] Garcia-Barceló MM, Yeung MY, Miao XP, Tang CSM, Cheng G, Chen G, et al. Genome-wide association study identifies a susceptibility locus for biliary atresia on 10q24.2. Human Molecular Genetics. 2010 Jul;19(14):2917–2925.

[242] Hirschfield GM, Liu X, Xu C, Lu Y, Xie G, Lu Y, et al. Primary biliary cirrhosis associated with HLA, IL12A, and IL12RB2 variants. The New England Journal of Medicine. 2009 Jun;360(24):2544–2555.

[243] Liu X, Invernizzi P, Lu Y, Kosoy R, Lu Y, Bianchi I, et al. Genome-wide meta-analyses identify three loci associated with primary biliary cirrhosis. Nature Genetics. 2010 Aug;42(8):658–660.

[244] Mells GF, Floyd JAB, Morley KI, Cordell HJ, Franklin CS, Shin SY, et al. Genome-wide association study identifies 12 new susceptibility loci for primary biliary cirrhosis. Nature Genetics. 2011 Mar;43(4):329–332.

[245] Nakamura M, Nishida N, Kawashima M, Aiba Y, Tanaka A, Yasunami M, et al. Genome-wide association study identifies TNFSF15 and POU2AF1 as susceptibility loci for primary biliary cirrhosis in the Japanese population. American Journal of Human Genetics. 2012 Oct;91(4):721–728.

[246] Chen PL, Shih SR, Wang PW, Lin YC, Chu CC, Lin JH, et al. Genetic determinants of antithyroid drug-induced agranulocytosis by human leukocyte antigen genotyping and genome-wide association study. Nature Communications. 2015 Jul;6:7633.

[247] Chu X, Pan CM, Zhao SX, Liang J, Gao GQ, Zhang XM, et al. A genome-wide association study identifies two new risk loci for Graves' disease. Nature Genetics. 2011 Aug;43(9):897–901.

[248] Khong JJ, Burdon KP, Lu Y, Leonardos L, Laurie KJ, Walsh JP, et al. Association of Polymorphisms in MACRO Domain Containing 2 With Thyroid-Associated Orbitopathy. Investigative Ophthalmology & Visual Science. 2016;57(7):3129–3137.

[249] Nakabayashi K, Tajima A, Yamamoto K, Takahashi A, Hata K, Takashima Y, et al. Identification of independent risk loci for Graves' disease within the MHC in the Japanese population. Journal of Human Genetics. 2011 Nov;56(11):772–778.

[250] Zhao SX, Xue LQ, Liu W, Gu ZH, Pan CM, Yang SY, et al. Robust evidence for five new Graves' disease risk loci from a staged genome-wide association analysis. Human Molecular Genetics. 2013 Aug;22(16):3347–3362.

[251] Feehally J, Farrall M, Boland A, Gale DP, Gut I, Heath S, et al. HLA has strongest association with IgA nephropathy in genome-wide analysis. Journal of the American Society of Nephrology: JASN. 2010 Oct;21(10):1791–1797.

[252] Gharavi AG, Kiryluk K, Choi M, Li Y, Hou P, Xie J, et al. Genome-wide association study identifies susceptibility loci for IgA nephropathy. Nature Genetics. 2011 Mar;43(4):321–327.

[253] Kiryluk K, Li Y, Scolari F, Sanna-Cherchi S, Choi M, Verbitsky M, et al. Discovery of new risk loci for IgA nephropathy implicates genes involved in immunity against intestinal pathogens. Nature Genetics. 2014 Nov;46(11):1187–1196.

[254] Li M, Foo JN, Wang JQ, Low HQ, Tang XQ, Toh KY, et al. Identification of new susceptibility loci for IgA nephropathy in Han Chinese. Nature Communications. 2015 Jun;6:7270.

[255] Yang C, Jie W, Yanlong Y, Xuefeng G, Aihua T, Yong G, et al. Genome-wide association study identifies TNFSF13 as a susceptibility gene for IgA in a South Chinese population in smokers. Immunogenetics. 2012 Oct;64(10):747–753.

[256] Yu XQ, Li M, Zhang H, Low HQ, Wei X, Wang JQ, et al. A genome-wide association study in Han Chinese identifies multiple susceptibility loci for IgA nephropathy. Nature Genetics. 2011 Dec;44(2):178–182.

[257] Kirino Y, Bertsias G, Ishigatsubo Y, Mizuki N, Tugal-Tutkun I, Seyahi E, et al. Genome-wide association analysis identifies new susceptibility loci for Behçet's disease and epistasis between HLA-B*51 and ERAP1. Nature Genetics. 2013 Feb;45(2):202–207.
