# Supplementary material for: Using hierarchical similarity to examine the genetics of Behçet’s disease
Source: BMC Res Notes. 2021 Sep 10;14:353. doi: 10.1186/s13104-021-05767-6 (PMC8434716; doi:10.1186/s13104-021-05767-6)
Supplement: Supplementary file 2 — Additional file 2: Figure S1 shows the results from the Jaccard Similarity run. [file 13104_2021_5767_MOESM2_ESM.pdf]

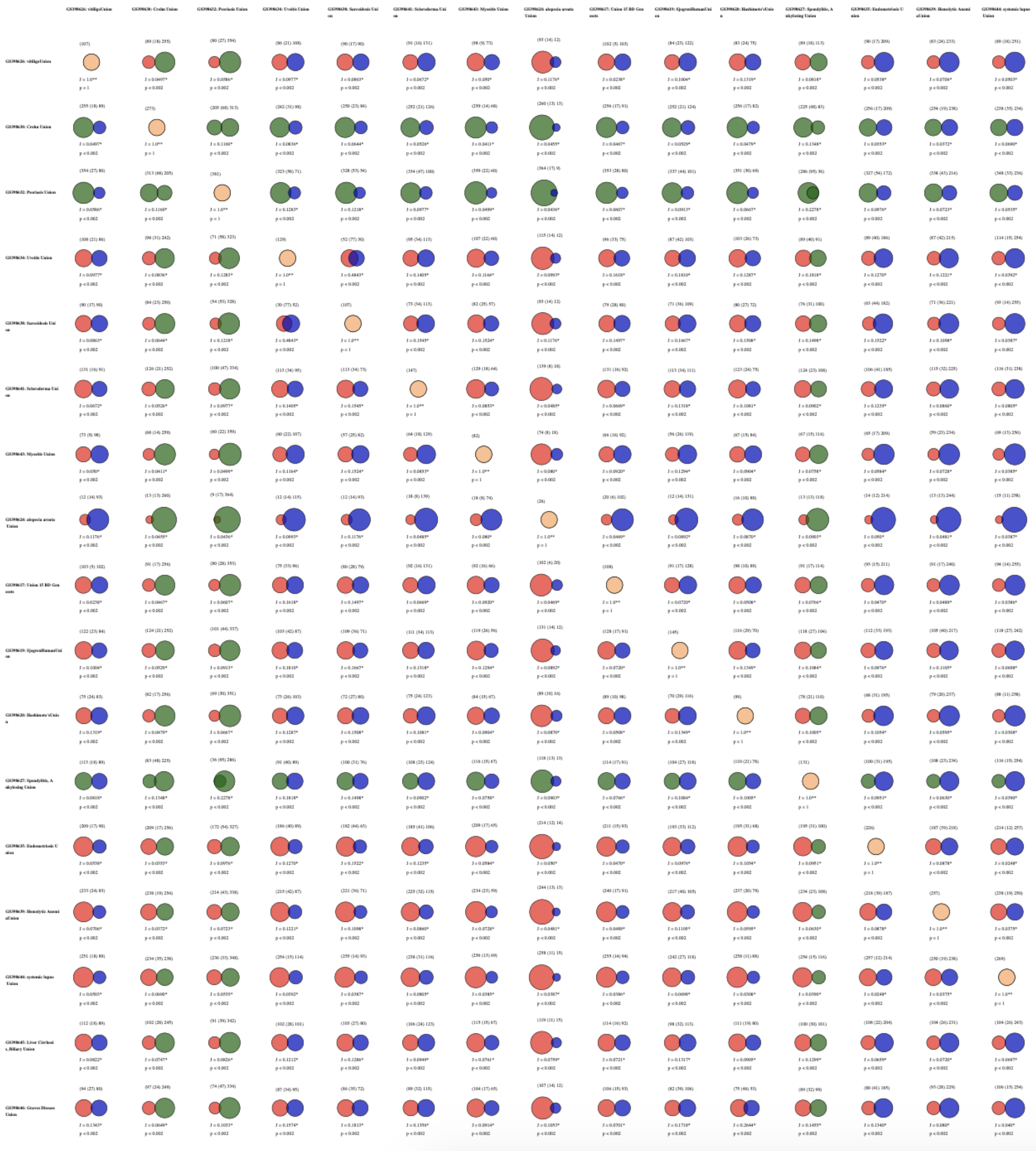

Figure S1: Results of the Jaccard Geneset Analysis. None of the 16 autoimmune disease genesets were identified as having a statistically significant overlap with BD.
